# SARS-CoV-2 hijacks p38β/MAPK11 to promote virus replication

**DOI:** 10.1128/mbio.01007-23

**Published:** 2023-06-22

**Authors:** Christina A. Higgins, Benjamin E. Nilsson-Payant, Boris Bonaventure, Andrew P. Kurland, Chengjin Ye, Tomer M. Yaron, Jared L. Johnson, Prithy Adhikary, Ilona Golynker, Maryline Panis, Oded Danziger, Brad R. Rosenberg, Lewis C. Cantley, Luis Martínez-Sobrido, Benjamin tenOever, Jeffrey R. Johnson

**Affiliations:** 1 Department of Microbiology, Icahn School of Medicine at Mount Sinai, New York, New York, USA; 2 Global Health and Emerging Pathogens Institute, Icahn School of Medicine at Mount Sinai, New York, New York, USA; 3 Department of Microbiology, New York University Langone Health, New York, New York, USA; 4 Vilcek Graduate School for Biomedical Sciences, New York University Langone Health, New York, New York, USA; 5 Texas Biomedical Research Institute, San Antonio, Texas, USA; 6 Meyer Cancer Center, Weill Cornell Medicine, New York, New York, USA; 7 Englander Institute for Precision Medicine, Institute for Computational Biomedicine Weill Cornell Medicine, New York, New York, USA; 8 Columbia University Vagelos College of Physicians and Surgeons, New York, New York, USA; 9 Department of Cell Biology, Harvard Medical School, Boston, Massachusetts, USA; 10 Dana-Farber Cancer Institute, Harvard Medical School, Boston, Massachusetts, USA; University of Pennsylvania, Philadelphia, Pennsylvania, USA

**Keywords:** p38 kinases, SARS-CoV-2, proteomics, phosphoproteomics, MAPK11, p38β

## Abstract

Severe acute respiratory syndrome coronavirus 2 (SARS-CoV-2), the causative agent of the coronavirus disease 2019 (COVID-19) pandemic, drastically modifies infected cells to optimize virus replication. One such modification is the activation of the host p38 mitogen-activated protein kinase (MAPK) pathway, which plays a major role in inflammatory cytokine production, a hallmark of severe COVID-19. We previously demonstrated that inhibition of p38/MAPK activity in SARS-CoV-2-infected cells reduced both cytokine production and viral replication. Here, we combined quantitative genetic screening, genomics, proteomics, and phosphoproteomics to better understand mechanisms underlying the dependence of SARS-CoV-2 on the p38 pathway. We found that p38β is a critical host factor for SARS-CoV-2 replication in multiple relevant cell lines and that it functions at a step after viral mRNA expression. We identified putative host and viral p38β substrates in the context of SARS-CoV-2 infection and found that most host substrates have intrinsic antiviral activities. Taken together, this study reveals a unique proviral function for p38β and supports exploring p38β inhibitor development as a strategy toward creating a new class of COVID-19 therapies.

**IMPORTANCE:**

SARS-CoV-2 is the causative agent of the COVID-19 pandemic that has claimed millions of lives since its emergence in 2019. SARS-CoV-2 infection of human cells requires the activity of several cellular pathways for successful replication. One such pathway, the p38 MAPK pathway, is required for virus replication and disease pathogenesis. Here, we applied systems biology approaches to understand how MAPK pathways benefit SARS-CoV-2 replication to inform the development of novel COVID-19 drug therapies.

## INTRODUCTION

Severe acute respiratory syndrome coronavirus 2 (SARS-CoV-2), the causative agent of the coronavirus disease 2019 (COVID-19) pandemic, has killed millions since it emerged in 2019. Severe COVID-19 cases are associated with excessive lung inflammation that can lead to acute respiratory distress syndrome, respiratory failure, multi-organ failure, and death (1, 2). This excessive inflammation is in part driven by an imbalanced immune response; compared to other respiratory virus infections, SARS-CoV-2 infection leads to excessive pro-inflammatory cytokine and chemokine production, with a delay in type I interferon (IFN-I) induction (3). While vaccines are highly effective at preventing severe illness and death, novel SARS-CoV-2 variants are continuously emerging with the ability to partially escape prior immunity. Currently, three antiviral therapies are available for COVID-19 treatment: remdesivir, molnupiravir, and Paxlovid (4 - 9). While effective, these therapies need to be administered early in infection, and as they all target single viral proteins, they are susceptible to the development of resistance. Host-directed therapies are attractive alternatives to antivirals as they are more likely to have broad antiviral activity and be less susceptible to resistance. One such therapy is dexamethasone, an immunomodulatory drug that combats inflammation and reduces COVID-19 mortality, bolstering the concept that targeting host pathways is a viable treatment strategy (10).

We previously reported that the p38 mitogen-activated protein kinase (p38/MAPK) pathway becomes activated during infection and that inhibition of p38 reduces both inflammatory cytokine expression and SARS-CoV-2 replication, suggesting that p38 inhibition may target multiple mechanisms related to SARS-CoV-2 pathogenesis (11). The p38 kinase family comprises four isoforms (α, β, γ, and δ) that are typically activated in concert by dual tyrosine and threonine phosphorylation by the MAP kinase kinases MAP2K3/MKK3 and MAP2K6/MKK6, but alternative and isoform-specific activation mechanisms have been described (12). Recent work mapped the activation of p38 in SARS-CoV-2 infection to viral entry, mediated by the viral spike protein (13). While the mechanisms by which the p38/MAPK pathway regulates inflammation are well described, the mechanism(s) by which it promotes SARS-CoV-2 replication is unknown. Furthermore, it is not known which kinase(s) at which levels of the p38/MAPK cascade impact SARS-CoV-2 replication (14).

Here we combined genetic and chemical perturbations with quantitative genomics, proteomics, and phosphoproteomics to better understand interactions between the p38/MAPK pathway and SARS-CoV-2 in human lung epithelial cell lines. We identified p38β as an essential host factor for SARS-CoV-2 replication in multiple cell lines and found that while p38β depletion did not impact viral mRNA abundance, it reduced the abundance of the viral nucleocapsid (N) protein and resulted in a significant induction of pro-inflammatory cytokines and IFN-I. We applied an unbiased approach to identify and test the impact of host and viral p38β substrates to provide a comprehensive view of the many factors regulating interactions between the p38 pathway and SARS-CoV-2 processes.

## RESULTS

### Comparisons across SARS-CoV-2 proteomics studies reveal pathways consistently regulated across species and cell types

**Fig 1:**
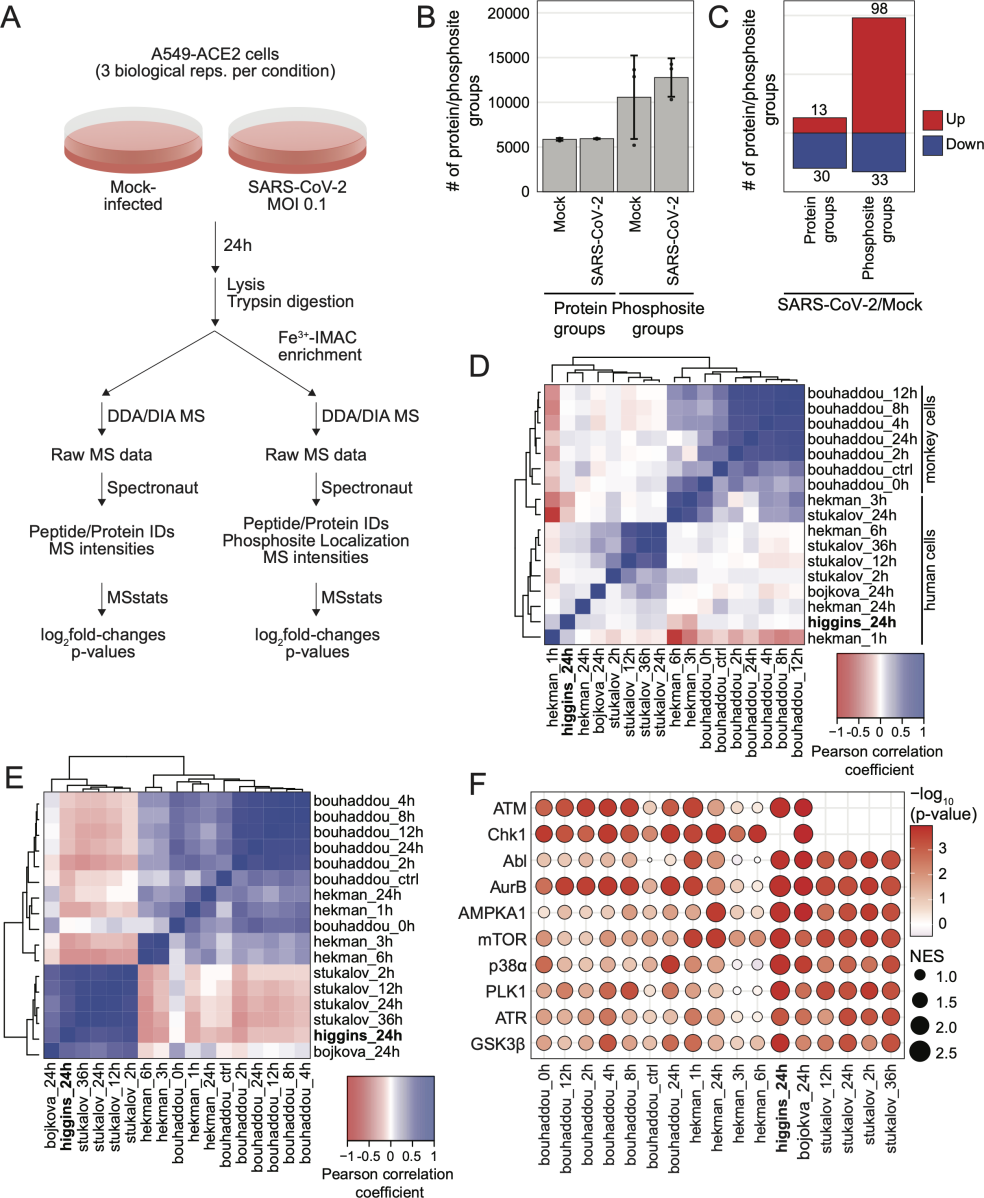
Comparisons across SARS-CoV-2 proteomics studies reveal pathways consistently regulated across species and cell types. (a) Schematic of experimental design; (b) plot of the average number of protein groups or phosphosite groups quantified in each condition; error bars represent 1 SD from the mean for three biological replicates; (c) plot of the number of differentially expressed protein groups or phosphosite groups for SARS-CoV-2-infected cells fold over mock-infected cells; significant change in abundance of protein group or phosphosite group defined as |log_2_(fold-change)| > 1 and *P*-value < 0.05, also see Table S1. (d) Heatmap of pairwise Pearson coefficients for phosphosite group log_2_(fold-change) profiles from this study and published studies indicated; (e) heatmap of pairwise Pearson coefficients based on log_10_(*P*-value) from kinase activity analysis of log_2_(fold-change) profiles from this study and published studies indicated, also see Table S2. (f) Bubble plot of kinase activity analysis of phosphosite group log_2_(fold-change) profiles for top 10 regulated kinases from this study and published studies indicated; the absolute value of the normalized enrichment score (NES) is indicated by node sizes and the −log_10_(*P*-value) is indicated by the color scale.

To better understand the host response to SARS-CoV-2 infection, we quantified changes in protein and phosphosite abundance in A549 human lung epithelial cells transduced to express ACE2 (A549-ACE2) infected with SARS-CoV-2 at a multiplicity of infection (MOI) of 0.1 for 24 hours (Fig. 1A). In total, this analysis comprised 6,089 unique protein groups and 16,032 unique phosphosite groups (Fig. 1B). Throughout this study, we considered protein/phosphosite groups with |log_2_fold-change| > 1 and *P*-value < 0.05 to be differentially abundant. At the protein level, SARS-CoV-2 proteins (N, ORF1AB/NSP1, ORF1AB/NSP3, ORF9B, and S) were increased by these criteria, with limited changes in host protein groups, consistent with SARS-CoV-2-mediated suppression of host protein synthesis (15). We observed more changes in the phosphoproteome, with 98 and 33 phosphosite groups increased and decreased, respectively (Fig. 1C; Table S1).

We next compared our A549 data (“higgins”) with four published proteomics studies of SARS-CoV-2 infection of the following cell types: Caco-2 human epithelial cells derived from a colorectal adenocarcinoma (“bojkova”), Vero E6 African Green Monkey kidney cells (“bouhaddou”), human-induced pluripotent stem cell-derived alveolar epithelial type 2 cells (iAT2, “hekman”), and A549 cells (“stukalov”) (11, 16 - 18). Pairwise Pearson correlation analysis of log_2_fold-change profiles for both protein abundance (Fig. S1A) and phosphorylation (Fig. 1D) data clustered primarily according to the animal species. Comparing data collected 24 hours post-infection in each study, four phosphosites were upregulated at least two-fold in four of these studies: HSPB1/HSP27 S15, MATR3 S188, TRIM28/TIF1B/KAP1 S473, and SZRD1 S107 (Table S2). None of these sites were detected in iAT2 cells. Two of these sites, HSPB1 S15 and TRIM28 S473, are p38/MAPK pathway substrates (19 - 21).

We next performed kinase activity analysis based on log_2_fold-change profiles using a gene set enrichment analysis (GSEA) approach with kinase-substrate annotations from PhosphoSite Plus (Fig. S1B; Table S3) (22 - 24). Human cell line kinase activity profiles were highly correlated (Fig. 1E). The 10 most regulated kinases across all data sets examined were AurB/AURKB, mTOR, Chk1, PLK1, GSK3β, p38ɑ, AMPKA1, ATM, ATR, and Abl (Fig. 1F). Kinases involved in cell cycle arrest, ATM, ATR, PLK1, and AurB, were regulated in all data sets, consistent with evidence that SARS-CoV-2 infection leads to cell cycle arrest (11). Of particular interest to this study, p38ɑ and its downstream kinase MAPKAP2 were significantly regulated in most studies compared (Fig. S1B; Table S3). We note that while all p38 isoforms are typically activated in parallel by upstream MAPKKs, p38 isoforms other than p38ɑ are less represented in the PhosphoSite Plus resource and are likely under-represented by this analysis.

### Multiple p38/MAPK pathway components impact SARS-CoV-2 infection in human lung epithelial cell lines

**Fig 2:**
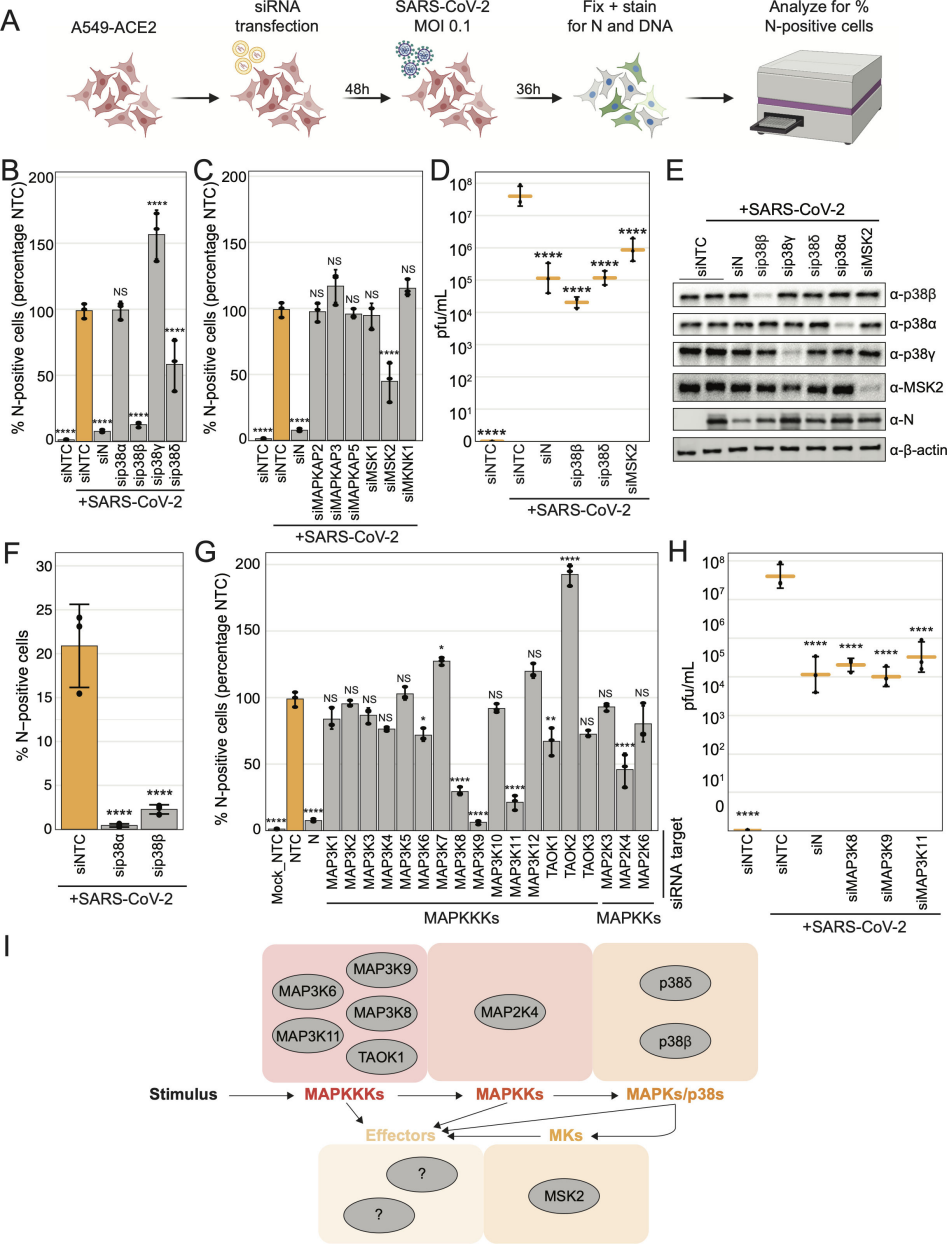
Multiple components of the p38/MAPK pathway impact SARS-CoV-2 infection in human lung epithelial cells. (a) Schematic of experiment workflow; (b and c) plots of the percent of SARS-CoV-2 N-positive cells analyzed using immunofluorescence cytometry, represented as a percentage of siNTC-transfected/SARS-CoV-2-infected control condition, for each indicated p38 isoform or downstream kinase siRNA transfection after SARS-CoV-2 infection at an MOI of 0.1 for 36 hours in A549-ACE2 cells; (d) plot of SARS-CoV-2 titers in plaque-forming units per milliliter (pfu/mL) from the supernatant collected from cells in 2b and c; (e) western blot of cell lysates collected in parallel with cells from 2b and c; (f) plot of the percent of SARS-CoV-2 N-positive cells after SARS-CoV-2 infection at an MOI of 0.15 for 24 hours in Calu-3 for each indicated siRNA transfection; (g) plot of the percent of SARS-CoV-2 N-positive cells, represented as a percentage compared to the siNTC-transfected/SARS-CoV-2-infected control condition, after SARS-CoV-2 infection at an MOI of 0.1 for 36 hours in A549-ACE2 for each indicated MAPKKK or MAPKK siRNA transfection; (h) plot of SARS-CoV-2 titers in pfu/mL in the supernatant collected from cells in 2g; (i) schematic of p38/MAPK pathway signal transduction highlighting proviral hits from 2b and 2e screens; black arrows indicate a possible phosphorylation event; all error bars represent 1 SD from the mean for three biological replicates; all *P*-value annotations were calculated using a one-way analysis of variance (ANOVA) test with *post hoc* testing using Tukey’s method comparing each condition with the control-infected condition for three biological replicates; “****” = *P*-value < 0.0001, “***” = 0.0001 < *P*-value < 0.001, “**” = 0.001 < *P*-value < 0.01, “*” = 0.01 < *P*-value < 0.05, “NS” = *P*-value > 0.05.

A gap remains in understanding which components of the p38/MAPK pathway impact SARS-CoV-2 replication. To address this, we employed small interfering RNA (siRNA) screening methodology to assess how the depletion of individual p38/MAPK pathway kinases affects SARS-CoV-2 replication. A549-ACE2 cells were transfected with siRNA pools targeting kinase genes, negative non-targeting control (NTC), or SARS-CoV-2 N positive control, and infected with 0.1 MOI of SARS-CoV-2 for 36 hours. The cells were then fixed and stained for SARS-CoV-2 N protein, and the percentage of N-positive cells was determined by immunofluorescence imaging cytometry and then normalized to the control-infected condition (siNTC-transfected and SARS-CoV-2 infected; Fig. 2A). We first screened the four p38 isoforms (p38ɑ/MAPK14, p38β/MAPK11, p38γ/MAPK12, and p38δ/MAPK13) as functional differences between the isoforms in the context of virus infection are particularly understudied. P38β depletion resulted in an approximate 90% reduction in infection, while depletion of p38ɑ did not affect infection in this cell type even though p38ɑ and p38β are often presumed to be functionally redundant, and p38ɑ is thought to be the major isoform regulating immune responses (25). We also found that p38δ depletion reduced infection by approximately 40% and that p38γ knockdown increased it by about 50% (Fig. 2B). Based on the mRNA-sequencing (mRNA-Seq) analysis of A549-ACE2 cells, p38ɑ is the most abundant isoform transcript, followed by p38γ, p38β, and lastly, p38δ (Fig. S2A). We then screened the kinases canonically downstream, MAPKAP2/MK2, MAPKAP3/MK3, MAPKAP5/MK5, MSK1, MSK2, and MKNK1, to test the hypothesis that downstream kinase(s) mediate the proviral activity of p38β. While MSK2 knockdown reduced infection by approximately 65%, depletion of the other downstream kinases had no effect (Fig. 2C). Next, compared to infected cells transfected with siNTC, knockdown of each putative proviral MAP kinase (p38β, δ, or MSK2) significantly decreased viral titer, up to 1,000-fold (Fig. 2D). We confirmed efficient siRNA knockdown of p38ɑ, p38β, p38γ, and MSK2 protein expression by western blotting (Fig. 2E) but were unable to verify knockdown of p38δ with commercial antibodies, likely because its basal expression is low in A549-ACE2 cells (Fig. S2A). Cell viability after siRNA transfection was decreased after p38β, p38δ, and MSK2 knockdown by 10%–20%, but it is unlikely that infection phenotypes observed are solely due to the decrease in cell viability because other targets such as MAPKAP3 also decreased cell viability but did not affect infection (Fig. S2B). To validate that the proviral p38β phenotype was not an off-target effect of the siRNAs, we replicated our findings with an independent set of controls and p38β gene-targeting pooled siRNAs (Fig. S2C and D). We next confirmed the effects of depletion of the p38ɑ and β isoforms in the Calu-3 epithelial lung adenocarcinoma cell line that expresses sufficient levels of endogenous ACE2 to facilitate SARS-CoV-2 infection. Depletion of p38ɑ and p38β reduced infection rates by approximately 98% and 90%, respectively, compared to NTC (Fig. 2F; Fig. S2E) and improved cell viability in mock-infected cells (Fig. S2F). Thus, while the effects of p38ɑ depletion were variable across cell lines, p38β depletion reduced SARS-CoV-2 infection rates consistently in all cell lines tested.

Focusing next on upstream portions of the p38/MAPK pathway, we tested individual MAPKKK or MAPKK knockdown on virus replication. Among the MAPKKKs screened, we found that MAP3K6/ASK2, MAP3K8/TPL2/COT, MAP3K9/MLK1, MAP3K11/MLK3, and TAOK1/MAP3K16 depletion reduced the percentage of SARS-CoV-2-infected cells by 30%–95%, while MAP3K7/TAK1 and TAOK2/MAP3K17/PSK knockdown increased it by nearly 30% and 100%, respectively (Fig. 2G). As for the MAPKKs, the canonical p38-regulating MAP2K3/MKK3 and MAP2K6/MKK6 had no phenotype, possibly because they are functionally redundant and may not exhibit a phenotype when knocked down individually (26). Interestingly, we found that depletion of MAP2K4/MKK4, widely considered a major regulator of JNK/MAPK signaling (24), decreased infection, indicating that JNK-mediated signaling may also impact SARS-CoV-2 replication. Of the upstream hits, cell viability was affected only by MAP3K8 knockdown (Fig. S2B). Finally, we confirmed that virus titers were significantly reduced on MAP3K8, MAP3K9, and MAP3K11 depletion (Fig. 2H). In summary, we found that SARS-CoV-2 replication is promoted by the p38/MAPK signaling cascade specifically involving, when tested individually, the MAPKKKs: MAP3K6, MAP3K8, MAP3K9, MAP3K11, and TAOK1; the MAPKK: MAP2K4; the p38s: p38β and p38δ; and the mediator kinase, MSK2 (Fig. 2I).

### p38β knockdown reduces viral protein, not viral mRNA, in human lung epithelial cells and promotes type I interferon activity

**Fig 3:**
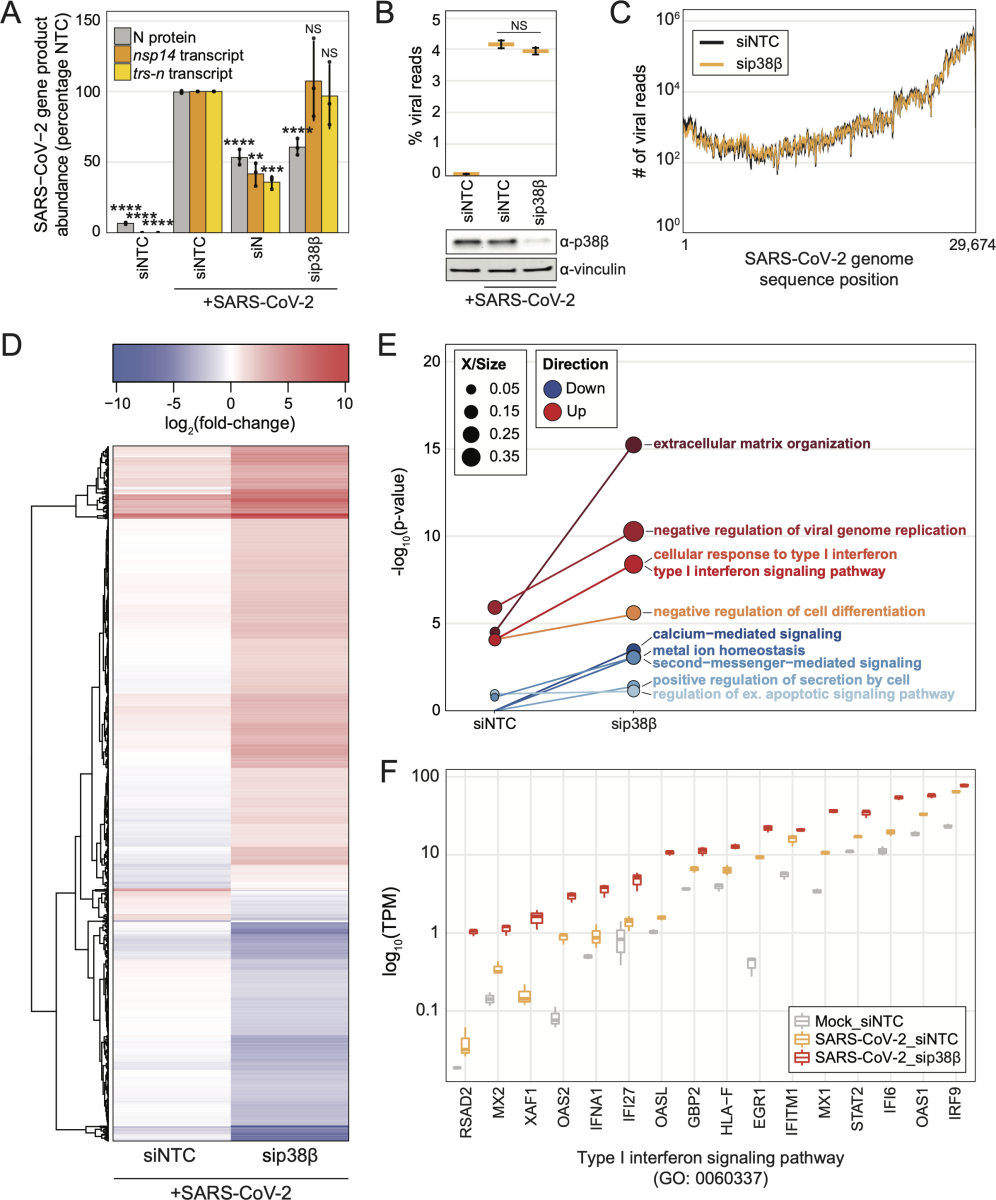
Analysis of cells infected with SARS-CoV-2 for a single virus life cycle reveals that p38β depletion reduces viral protein abundance, but not viral RNA abundance, and promotes type I interferon activity. (**a**) Plot of the percentage of SARS-CoV-2 N protein-positive cells analyzed using immunofluorescence microscopy (gray) or *nsp14* or *trs-n* transcript abundance detected using reverse transcriptase quantitative PCR (RT-qPCR) (gold/yellow) normalized to the control-infected condition after SARS-CoV-2 infection at an MOI of 2 for 8 hours in A549-ACE2 cells; error bars represent 1 SD from the mean; *P*-values were calculated using a one-way ANOVA test with *post hoc* testing using Tukey’s method comparing each condition to the control-infected condition; “****” = *P*-value < 0.0001, “***” = 0.0001 < *P*-value < 0.001, “**” = 0.001 < *P*-value < 0.01, “*” = 0.01 < *P*-value < 0.05, “NS” = *P*-value > 0.05; statistics for protein generated using three biological replicates and statistics for mRNA abundance generated using nine biological replicates; (**b**) plot of percent viral reads from mRNA-Seq of A549-ACE2 cells transfected with siNTC or sip38β and infected with SARS-CoV-2 MOI of 0.75 or mock-infected for 8 hours; significance annotated as *P*-value from one-way ANOVA test with *post hoc* testing using Tukey’s method comparing each condition to the control-infected condition; NS = *P*-value > 0.05; western blot from lysates collected in parallel below; (**c**) number of viral reads at each nucleotide in SARS-CoV-2 genome from mRNA-Seq; (**d**) hierarchically clustered heatmap of differentially expressed genes for sip38β-transfected/SARS-CoV-2-infected compared to siNTC-transfected/SARS-CoV-2-infected, but shown here as each infected condition fold over siNTC-transfected/mock-infected; rows represent each gene; color corresponds to log_2_(fold-change) as indicated; (**e**) plot of four most significant GO terms enriched from sip38β-transfected/SARS-CoV-2-infected fold over siNTC-transfected/SARS-CoV-2-infected differentially upregulated (red shades) or downregulated (blue shades) genes; point size represents proportion of total genes associated with a GO term; (**f**) plot of log_10_(transcripts per million [TPM]) for each gene represented in the indicated GO term for each condition, from same analysis as 3D. All raw and processed mRNA-Seq data are available on NCBI GEO GSE183999 (https://www.ncbi.nlm.nih.gov/geo/geo2r/?acc=GSE183999).

To begin characterizing p38β activity during infection, viral subgenomic RNA (sgRNA), genomic RNA (gRNA), and protein abundance were measured after a high MOI, single-cycle SARS-CoV-2 infection in A549-ACE2 cells transfected with siRNAs targeting controls or p38β. A single-cycle infection allowed us to observe the behavior of the virus during one life cycle without multiple iterations of infections confounding the results. First, as a control, the knockdown of SARS-CoV-2 N compared to NTC resulted in significant decreases in both viral protein and transcript abundance. P38β depletion resulted in no change in gRNA (i.e., *nsp14*) or subgenomic mRNA (i.e., *trs-n*) abundance; however, knockdown of p38β resulted in a significant decrease in viral protein (Fig. 3A). These findings suggest the mechanism by which p38β promotes virus replication acts after viral RNA synthesis.

We next used mRNA sequencing to analyze transcriptome changes in p38β-depleted or control A549-ACE2 cells infected for a single replication cycle of SARS-CoV-2. Samples clustered by condition as measured by principal component analysis (Fig. S3A and B). Control-infected cells fold over mock-infected cells resulted in 197 differentially expressed genes (DEGs, |log_2_fold-change| > 1.5 and adjusted *P*-value < 0.05) and p38β-depleted, infected cells fold over mock-infected cells resulted in 1,303 DEGs (Fig. S3C and D; Table S4). Consistent with the reverse transcriptase quantitative PCR (RT-qPCR) results, the percentage of viral reads and the number of viral reads at each position in the viral genome did not change between control-infected cells and p38β-depleted cells (Fig. 3B and C).

937 genes were differentially expressed between the infected, p38β-depleted cells, and control-infected cells (Table S4). These genes, shown as a heatmap of log_2_fold-changes for each condition fold over mock (Fig. 3D), frequently trended in the same direction, but to a larger degree with p38β knockdown. Additionally, gene ontology (GO) enrichment analysis revealed that GO terms related to extracellular matrix organization and IFN-I were significantly enriched by the upregulated DEGs (Fig. 3E). These data suggest that p38β negatively regulates the expression of pro-inflammatory cytokines and IFN-I, which was not expected as p38 kinases are generally thought to positively regulate cytokine expression. The same analysis on the downregulated DEGs enriched for GO terms related to second messenger–mediated signaling, metal ion homeostasis, and cell secretions (Fig. 3E; Table S5). Focusing on the genes that contributed to the significance of the “Type I interferon signaling pathway” term, some genes were impacted more than others by p38β depletion compared to control-infected and mock-infected conditions. For example, the transcripts per million (TPM) read counts for genes such as *RSAD2*, *IFNA1, IFI27*, and *OASL* were similar for mock-infected and control-infected conditions but much higher in the p38-depleted condition, whereas for most other genes, both infected conditions had more counts than the mock (Fig. 3F). These data suggest p38β may differentially regulate the expression of IFN-related genes in this biological context. Genes that contributed to the most significantly enriched annotations, extracellular matrix organization, and calcium-mediated signaling exhibited similar abundance changes (Fig. S3E and F).,

### p38β proviral mechanism is primarily STAT1-independent but leads to ISG expression as a byproduct

**Fig 4:**
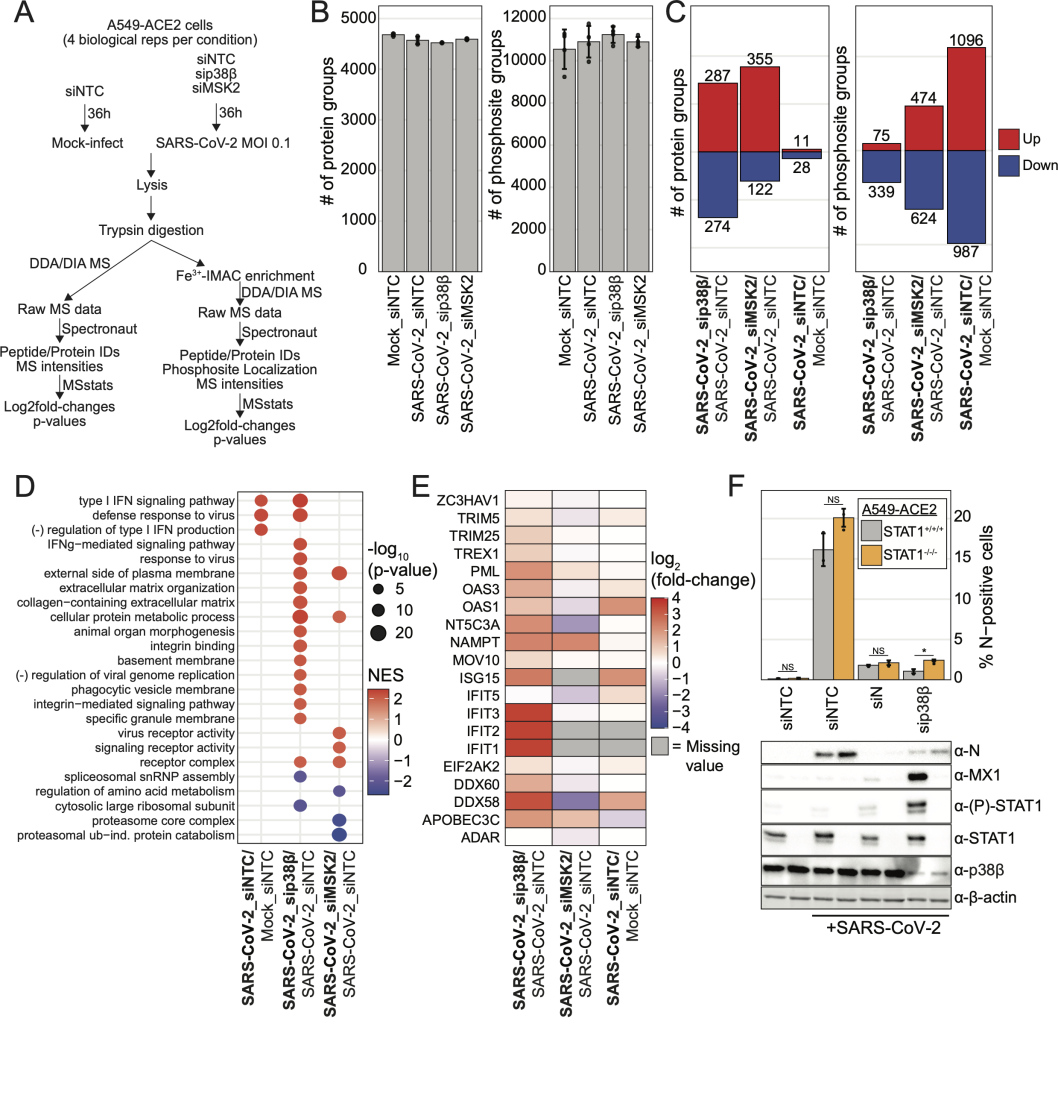
p38β proviral mechanism is primarily STAT1-independent but leads to ISG expression as a byproduct. (a) Schematic of experiment workflow; (b) plot of the number of protein groups or number of phosphosite groups identified in each biological replicate; error bars represent 1 SD from the mean for four biological replicates; (c) plot of the number of significantly differentially abundant protein groups and phosphosite groups for condition comparisons as indicated; significant change in abundance of protein group or phosphosite group defined as |log_2_(fold-change)| > 1 and *P*-value < 0.05; (d) GO terms enriched from he analysis of differentially abundant protein groups for each comparison from protein abundance data using gene set enrichment analysis (GSEA); (e) heatmap of log_2_(fold-change) for each indicated ISG in each indicated condition comparison from protein abundance data; (f) plot of the percent of SARS-CoV-2 N-positive cells analyzed using immunofluorescence cytometry for each indicated transfection condition after SARS-CoV-2 infection at an MOI of 0.1 for 30 hours in A549-ACE2 cells or A549-ACE2ΔSTAT1; error bars represent 1 SD from the mean for three biological replicates; *P*-value annotations were calculated using a one-way ANOVA test with *post hoc* testing using Tukey’s method comparing each condition between each cell type for three biological replicates; “****” = *P*-value < 0.0001, “***” = 0.0001 < *P*-value < 0.001, “**” = 0.001 < *P*-value < 0.01, “*” = 0.01 < *P*-value < 0.05, “NS” = *P*-value > 0.05; below are western blots of lysates collected in parallel with cells analyzed in above plot.

To assess if transcriptome changes were also reflected at the proteome level, we assessed how siRNA knockdown of p38β or MSK2 in the context of SARS-CoV-2 infection affects the host proteome using quantitative proteomics. In biological quadruplicate, A549-ACE2 cells were transfected with pooled siRNAs targeting NTC, p38β, or MSK2. Cells were then infected with SARS-CoV-2, and 36 hours post-infection, cells were lysed and subjected to quantitative proteome and phosphoproteome analysis (Fig. 4A). A total of 4,900 unique protein groups and 14,414 unique phosphosite groups were identified (Fig. 4B; Table S1). These data clustered by their respective siRNA targets in Pearson correlation analysis, and each sample had similar normalized log_2_intensity distributions (Fig. S4A through D). In comparison to siNTC-transfected/mock-infected cells, siNTC-transfected/SARS-CoV-2-infected (control-infected) cells yielded few changes to the proteome and large changes to the phosphoproteome with approximately 1,000 phosphosite groups significantly changing (Fig. 4C). More changes were observed here than in our preliminary A549-ACE2 analysis (Fig. 1); the siRNA analysis included a greater number of biological replicates (4 versus 3) and reduced technical variability that yielded a greater number of features with lower *P*-values. Furthermore, siRNA transfection may have impacted the number of significant changes.

Knockdown of each kinase in SARS-CoV-2-infected cells led to substantial changes to the proteome. Compared to control-infected cells, knockdown of p38β or MSK2 led to 287 and 355 unique protein groups significantly increasing, respectively, and 274 and 122 protein groups significantly decreasing, respectively. There were also significant changes to the phosphoproteome; in comparison to control-infected cells, knockdown of p38β or MSK2 led to 75 and 474 unique phosphosite groups significantly increasing, respectively, and 339 and 624 phosphosite groups significantly decreasing, respectively (Fig. 4C; Table S1). Consistent with our observations made at the transcriptome level, GO enrichment analysis of protein abundance log_2_fold-change profiles revealed that SARS-CoV-2-infected cells depleted of p38β exhibited a strong IFN-I signature compared to control-infected cells. MSK2 knockdown in SARS-CoV-2-infected cells did not lead to a comparable phenotype, suggesting MSK2 does not mediate p38β-related interferon regulation (Fig. 4D; Table S6). Focusing on well-characterized ISGs (27), most were enhanced in response to p38β depletion compared to control-infected cells (Fig. 4E). Western blotting confirmed that p38β knockdown led to an increase in MX1, a prototypical ISG, and specifically in the context of infection (Fig. S4E).

These findings led us to question whether perturbation of p38β prevents SARS-CoV-2 replication by inducing ISG expression, which in turn suppresses viral replication, or if perturbation of p38β prevents replication in an independent manner that incidentally leads to the expression of ISGs, for example, by exposing a pathogen-associated molecular pattern (PAMP) that is detected by the innate immune sensors. To address this, we performed SARS-CoV-2 infections and control or p38β siRNA knockdowns in A549-ACE2 cells or STAT1-knockout A549-ACE2 cells, which are insensitive to IFN-I and IFN-III signaling. If STAT1-dependent expression of ISGs is required to restrict infection in cells with reduced p38β expression, deletion of STAT1 would restore SARS-CoV-2 replication to wild-type levels. We found that while the reduction in infection when p38β is depleted is significant, it is not appreciably rescued by STAT1-knockout, indicating that the mechanism of action of p38β is primarily STAT1-independent. We confirmed by western blotting efficient knockdown of p38β, STAT1-knockout, ablation of MX1 expression, and STAT1 phosphorylation in the STAT1-knockout A549-ACE2 cells (Fig. 4F). We next tested the JAK1/2 inhibitor ruxolitinib, which acts upstream of STAT1 and is broadly effective at preventing IFN (and another cytokine) signaling, and again observed that JAK1/2 inhibition did not rescue the infection defect associated with p38β knockdown (Fig. S4F). These findings demonstrate that the enhanced antiviral response that results from p38β knockdown is not the primary mechanism by which SARS-CoV-2 infection is reduced.

### Quantitative, unbiased phosphoproteomics analysis pipeline identifies novel putative p38β substrates

**Fig 5:**
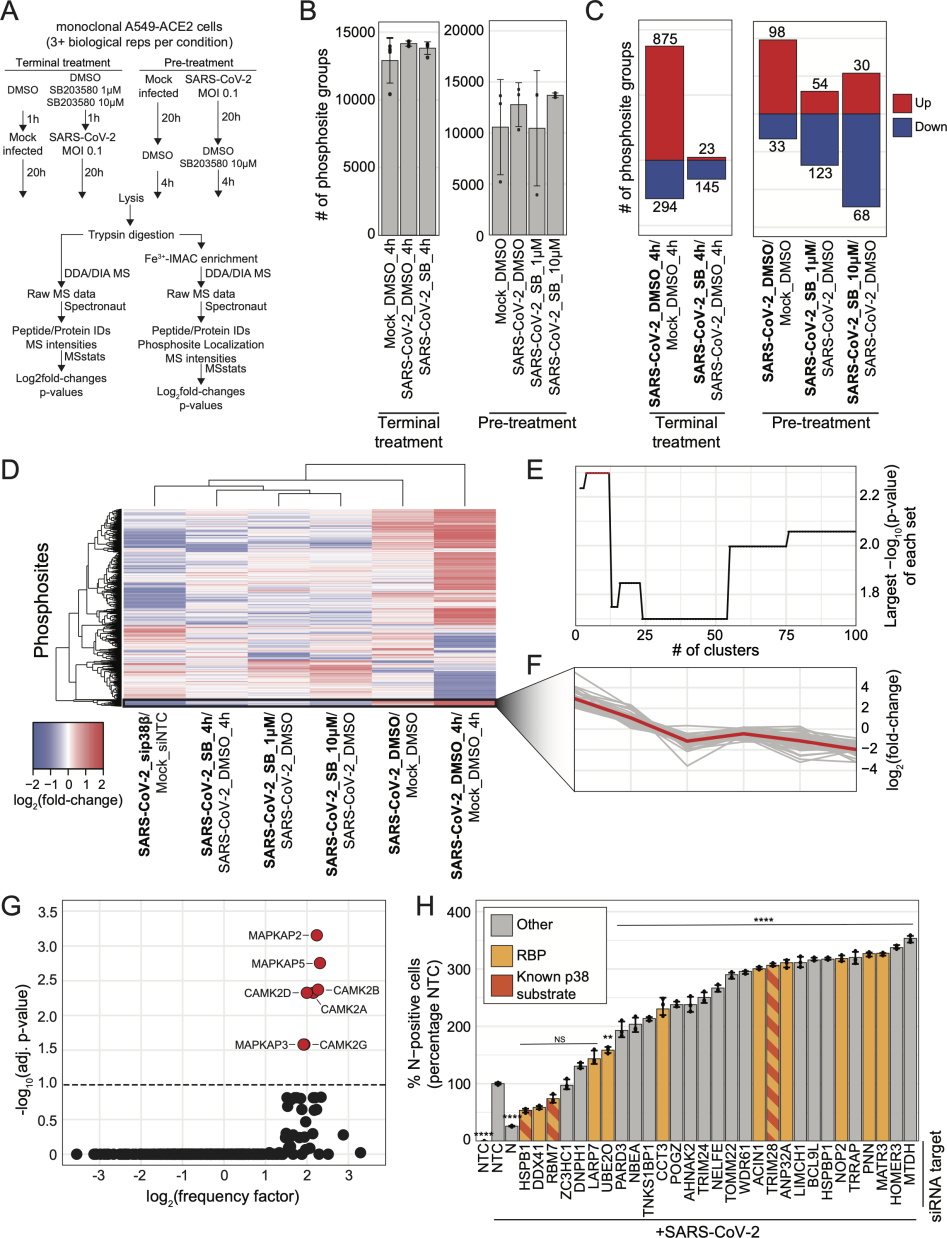
Novel, putative p38 pathway kinase substrates identified using kinase perturbation phospho-profile clustering (KiPPC) analysis pipeline. (a) Schematic of experiment workflow; (b) plot of the number of phosphosite groups identified in each condition; error bars represent 1 SD from the mean for at least three biological replicates; (c) plot of the number of significantly differentially abundant phosphosite groups for each indicated condition comparison for the “terminal treatment” experiment arm (left) or the “pre-treatment” experiment arm (right); significant change in abundance of protein group or phosphosite group defined as |log_2_(fold-change)| > 1 and *P*-value < 0.05; (d) heatmap of log_2_(fold-change) for differentially abundant phosphosite groups (rows) in each indicated condition comparison (columns), hierarchically clustered; cluster of interest in black box; (e) plot of -log_10_(*P*-value) of each highest −log_10_(*P*-value) cluster from each clustering iteration; (f) plot of log_2_(fold-change) profiles of cluster-of-interest phosphosite groups (gray line) for each condition comparison in 5D; red line indicates average profile for all cluster-of-interest phosphosite groups; (g) plot of −log_10_(adjusted *P*-value) and −log_2_(frequency factor) based on the comparison of cluster-of-interest phosphosite motif sequences to the consensus substrate motif sequence for each characterized human kinase; red points are kinases with significantly similar consensus substrate motifs to the cluster-of-interest phosphosite group sequences, and black points represent kinases with insignificantly similar consensus substrate motifs; (h) plot of percent of SARS-CoV-2 N-positive cells analyzed using immunofluorescence cytometry normalized to the control-infected condition for each indicated transfection condition after SARS-CoV-2 infection at an MOI of 0.1 for 30 hours in A549-ACE2 cells; error bars represent 1 SD from the mean for three biological replicates; *P*-values were calculated using a one-way ANOVA test with *post hoc* testing using Tukey’s method comparing each condition to the control-infected condition for three biological replicates; “****” = *P*-value < 0.0001, “***” = 0.0001 < *P*-value < 0.001, “**” = 0.001 < *P*-value < 0.01, “*” = 0.01 < *P*-value < 0.05, “NS” = *P*-value > 0.05.

To identify putative p38β substrates that may explain the mechanism by which p38β promotes infection, we created an analysis pipeline to assess proteome and phosphoproteome data from experiments employing three different p38β perturbation strategies: (1) siRNA knockdown of p38β (described previously), (2) titrated treatment of cells with the p38ɑ/β inhibitor SB203580 beginning 1 hour before a 24-hour SARS-CoV-2 infection (pre-treatment), and (3) a 24-hour SARS-CoV-2 infection with the last four hours being in the presence of SB203580 (terminal treatment) (Fig. 5A).

For the chemical perturbation strategies, we selected SB203580, a well-characterized p38ɑ/β-specific small molecule inhibitor, as p38β-selective inhibitors are not currently available (28). However, SB203580 is estimated to be 10 times more potent at inhibiting p38ɑ as p38β (29). These data clustered by their respective conditions in Pearson correlation analysis, and each sample had similar normalized log_2_intensity distributions within each experiment (Fig. S4A through H). The terminal drug treatment experiment yielded 16,220 unique phosphosite groups, and the pre-treatment experiment, 16,032 (Fig. 5B; Table S1). Terminally treated samples had substantially fewer changes in the proteome and phosphoproteome compared to the pre-treated samples (Fig. 4C), likely reflecting how the limited drug-exposure time does not allow for significant changes in protein expression, and how pre-treatment significantly affects infection (Fig. S4I; Table S1). Confirming successful drug treatment, we observed a significant decrease in the phosphorylation of known p38 substrates (PARN S557, RIPK1 S320, CP131 S47, and HSPB1 S15) in response to SB203580 in both pre-treatment and terminal treatment experiments (Fig. S5J and K). In the pre-treatment protein abundance data, we did not see an upregulation of ISGs, contrasting with observations made on genetic perturbation of p38β (Table S1). We hypothesize that the ISG phenotype did not develop because, as SB203580 is primarily a p38ɑ inhibitor, the inhibition of p38β by SB203580 was not as effective as genetic inhibition.

Proceeding with our analysis pipeline, we next combined the phosphoproteome profiles of both drug treatment data sets with the p38β knockdown profiles and developed a supervised hierarchical clustering approach called kinase perturbation phospho-profile clustering (KiPPC) (Fig. S5L). Data were first filtered for singly phosphorylated phosphosite groups, with no missing values across comparisons and significant changes in at least one comparison, yielding 1,191 total phosphosite profiles, including 12 phosphosites annotated in Phosphosite Plus as substrates of p38α, p38β, or one of their downstream effector kinases (i.e., MAPKAP2, MAPKAP3, MAPKAP5, MSK1, MSK2, or MKNK1) (23). The profiles were then hierarchically clustered based on their Euclidean distances (Fig. 5D), generating a dendrogram tree that was then cut iteratively 99 times in the order of decreasing height to generate between 2 and 100 clusters. For each iteration, the significance of the enrichment of p38α/β substrates in each cluster was calculated with a hypergeometric test. The cluster most significantly enriched for known p38α/β substrates occurred in iterations 3–12, with a hypergeometric *P*-value of 0.005 (Fig. 5E). The phosphosite profiles within this cluster are very similar, with the representative profile behaving as expected: during SARS-CoV-2 infection, the log_2_fold-change is high because the p38 pathway is active, and when p38β is genetically or chemically inhibited, it decreases (Fig. 5F). The cluster contains 35 phosphosites in total including three annotated p38α/β substrate sites (HSPB1 S15, RBM7 S137, and TRIM28 S473), several p38α/β substrates at phosphosites not previously annotated as p38α/β-dependent (TRIM28 S471, HSPB1 S78, HSPB1 S82, NELFE S51), as well as proteins physically associated with annotated p38α/β substrates (LARP7 and TRIM24) (Table S7). Additionally, the cluster is enriched for processes commonly associated with the p38/MAPK pathway including RNA binding, protein folding, and transcription elongation (Fig. S5M) (14). In support of our KiPPC analysis results, we used the Kinase Library to analyze our cluster’s phosphosite motifs and found that the kinases most likely to phosphorylate these substrates are p38/MAPK pathway members, MAPKAP2, MAPKAP3, and MAPKAP5, and related CAMK-type kinases, CAMK2-A, -B, -D, and -G (Fig. 5G) (30). We next aimed to determine if any of the novel, putative substrates impacted SARS-CoV-2 replication by employing the same siRNA screening methodology described previously. We found that depletion of a vast majority of putative p38α/β substrates tested (22 of 29) resulted in a significant increase in the percentage of N-positive cells (Fig. 5H). We also assessed cell viability in response to each siRNA transfection (Fig. S5N). While these data do not specifically reflect the impact of phosphorylation at these sites on virus replication, this screen revealed that a large number of putative p38α/β substrates play critical roles in SARS-CoV-2 infection.

### SARS-CoV-2 N protein phosphorylation is sensitive to p38 inhibition

**Fig 6:**
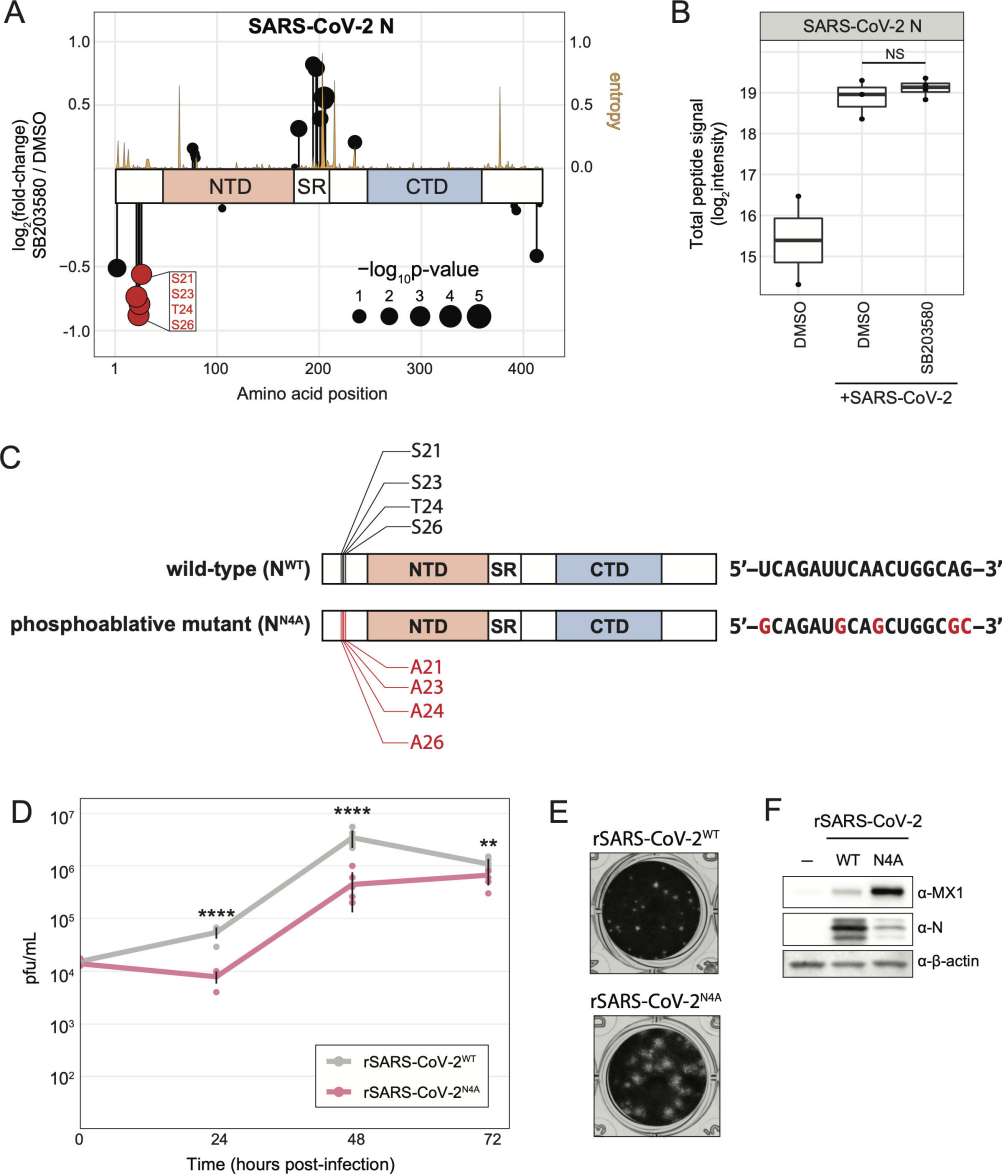
Phosphoablative mutation of four SB203580-sensitive residues on SARS-CoV-2 nucleocapsid protein attenuates virus growth. (a) Plot of log_2_(fold-changes) for each phosphosite group on SARS-CoV-2 N differentially abundant for 10 µM SB203580-terminally treated/SARS-CoV-2-infected fold over control-infected condition; entropy (amino acid sequence conservation between SARS-CoV-2 variants; higher entropy = less conserved) indicated as yellow line; (b) plot of log_2_(signal intensity) of total N protein abundance from terminal treatment experiment (Fig. 5A); NS = *P*-value > 0.05; (c) schematic comparing N^WT^ to N^N4A^ (left), and the corresponding nucleotide sequence conferring the mutation (right); (d) graph comparing rSARS-CoV-2^WT^ and rSARS-CoV-2^N4A^ titer (pfu/mL) at several points during an infection performed at an MOI of 0.01 in A549-ACE2 cells; error bars represent 1 SD from the mean for six biological replicates; *P*-values were calculated using a two-tailed Student’s *t*-test for six biological replicates; “****” = *P*-value < 0.0001, “***” = 0.0001 < *P*-value < 0.001, “**” = 0.001 < *P*-value < 0.01, “*” = 0.01 < *P*-value < 0.05, “NS” = *P*-value > 0.05; (e) Image of crystal violet-stained wells from plaque assay of infected A549-ACE2 cell supernatant performed with Vero E6 cells; (f) western blots of lysates from A549-ACE2 cells infected with the indicated SARS-CoV-2 variant at an MOI of 0.1 for 24 hours.

In addition to identifying novel host p38 substrates, we also explored the possibility of p38-dependent phosphorylation of SARS-CoV-2 proteins. To focus on viral phosphosites, we specifically looked at the short, terminal drug treatment data set because this experimental framework does not affect total viral protein abundance, allowing us to directly and accurately quantify changes to viral phosphosites in response to p38 inhibition. In our analyses, SARS-CoV-2 N was the only viral protein identified that harbored SB203580-sensitive phosphosites based on *P*-values, although the fold-changes were less than the two-fold threshold we implemented throughout this study. We identified four N phosphosites (S21, S23, T24, and S26) that decreased during infection in response to SB203580 compared to DMSO treatment (Fig. 6A; Fig. S6A). These sites are located in an intrinsically disordered region close to the *N*-terminus of N. Additionally, these amino acid residues have no or relatively low entropy (i.e., variation) among SARS-CoV-2 variants in the Nextstrain resource (Fig. 6A) (31). We confirmed no significant difference in total N protein abundance between DMSO-treated, infected cells and SB203580-treated, infected cells (Fig. 6B). These sites, phosphorylated either directly or indirectly by p38ɑ and/or p38β, could confer changes in N activity, affecting virus replication. To test this hypothesis, we generated a recombinant, phosphoablative mutant of SARS-CoV-2 USA-WA1/2020 (rSARS-CoV-2^N4A^) containing alanine substitutions at the four SB203580-sensitive phosphoresidues (Fig. 6C). In a time course experiment comparing virus titer of recombinant wild-type SARS-CoV-2 USA-WA1/2020 (rSARS-CoV-2^WT^) with rSARS-CoV-2^N4A^, the mutant virus was significantly attenuated in titer at several timepoints (Fig. 6D). Interestingly, we also observed that rSARS-CoV-2^N4A^ infection of Vero E6 produced morphologically different plaques, larger with less defined edges, than rSARS-CoV-2^WT^ (Fig. 6E). Lastly, rSARS-CoV-2^N4A^ infection of A549-ACE2 cells led to a higher induction of a canonical ISG, MX1, than infection with rSARS-CoV-2^WT^ (Fig. 6F); this observation could be a result of altering the viral gene ORF9B that overlaps with N. In summary, we found that ablation of the phosphorylation of *N*-terminal N residues inhibited viral production, albeit less significantly than genetic inhibition of p38β, suggesting that p38 impacts viral replication by modulating both viral and host substrates.

## DISCUSSION

The p38/MAPK pathway responds to and regulates the production of inflammatory cytokines and chemokines via effector kinases, altering the activities of transcription factors, RNA-binding proteins, and translation factors (14). For several viral pathogens including Dengue virus, SARS-CoV, and influenza A virus, inhibition of p38/MAPK activity reduced inflammatory cytokine production, led to better control of the infection, and increased survival in mice (32 - 34). However, p38/MAPK inhibition did not directly impair the replication of these viruses. In contrast, we demonstrated that depletion of p38/MAPK components blocked SARS-CoV-2 replication.

Here, we screened MAPK components to identify the MAPKKK, MAPKK, MAPKs, and downstream kinases that impact SARS-CoV-2 infection. We found that p38β depletion greatly reduced SARS-CoV-2 infection rates in two lung epithelial cell lines, A549-ACE2 and Calu-3, while p38α depletion only impacted infection in Calu-3 cells. We only analyzed single knockdowns, but it is possible that there are additional components of the p38/MAPK pathway regulating SARS-CoV-2 infection that were masked by functional redundancy in our screen. P38α is presumed to be the major isoform involved in inducing immune responses, as p38β knockout in mice contributes to neither p38-dependent immediate-early gene transcription nor lipopolysaccharide (LPS)-induced inflammation. However, while immune responses in p38β^-/-^ mice have been assessed in the contexts of LPS stimulation and tumor necrosis factor overexpression, they have not been assessed in the context of viral infection (25). Our findings emphasize the need for further research and reagents to help better characterize p38β. P38β does not appear to be essential as p38β knockout mice are viable and fertile, whereas p38ɑ knockout mice are embryonically lethal, and as p38β has a distinctly smaller active site than p38ɑ, the development of specific inhibitors is conceivable (25). Thus, p38β makes an attractive target for the treatment of COVID-19. Additional studies are needed to validate these findings *in vivo* and to determine if p38β is important for the replication of other coronaviruses and other virus families.

Proteomics applications require large amounts of cells; thus, this study was limited to lung epithelial cell lines that may differ from *in vivo* responses. However, recent work in ex vivo human lung explants and lung epithelial (AT-II) organoids demonstrated a reduction in inflammatory cytokine production in response to p38/MAPK inhibition (13). While that study did not reveal inhibition of SARS-CoV-2 infection in response to p38/MAPK inhibition, both inhibitors used throughout the study are 4- to 14-fold more selective for p38ɑ than p38β. No p38β-selective inhibitors are currently available, but an analog-sensitive kinase approach has been demonstrated for p38 kinases previously and could be applied to distinguish p38ɑ from p38β activity in the context of SARS-CoV-2 processes (35).

Quantitative phosphoproteomics uncovered novel, putative host and viral p38 substrates in the context of SARS-CoV-2 infection. We found a cluster of phosphorylation sites on the SARS-CoV-2 N protein that were sensitive to p38 inhibition and, when substituted with phosphoablative alanine residues, reduced SARS-CoV-2 replication and activated the IFN pathway. While our study revealed that these phosphorylation sites are sensitive to p38 inhibition, the sequence flanking these residues does not match predicted p38 sequence preferences. P38 kinases prefer to phosphorylate residues preceded by a proline, which is not true for these p38 inhibitor-sensitive N phosphorylation sites. Rather, we predict that N phosphorylation is an indirect effect in response to p38/MAPK activation.

Throughout the virus life cycle, the coronavirus N protein oligomerizes along the length of the viral RNA allowing enhanced viral polymerase activity, template switching, and innate immunity evasion (36). N post-translational modifications have been documented to affect N activities; avian *Gammacoronaviru*s infectious bronchitis virus N phosphorylation increases the affinity of N for viral RNA compared to non-viral RNA (37). Additionally, *Betacoronavirus* murine hepatitis virus N phosphorylation by GSK-3 promotes the synthesis of genomic RNA over subgenomic RNA by promoting template read-through (38). Specific to SARS-CoV-2, many studies have implicated SARS-CoV-2 N phosphorylation in processes including liquid–liquid phase separation and innate immunity activation, but mechanisms to explain phenotypes have yet to be elucidated (39 - 41). In this study, we identified phosphosites on SARS-CoV-2 N that are sensitive to the p38α/β inhibitor SB203580. As N phosphorylation is known to affect its activity, it is plausible that p38-dependent N phosphorylation is responsible for the phenotypes we observed, but we cannot exclude the possibility that p38-dependent phosphorylation of host protein(s) may play a more significant role in driving these phenotypes.

Finally, our study combined genetic and chemical perturbations of p38 to identify host substrates in the context of SARS-CoV-2 infection. An siRNA screen of putative p38 substrates revealed a strong enrichment for genes with antiviral activity (depletion of 22 of 29 genes tested significantly increased infection rates). Several of these novel substrates have been previously implicated as relevant in the context of SARS-CoV-2 infection; single nucleotide polymorphisms (SNPs) in their respective phosphosites are naturally occurring in the human population, and some SNPs have been associated with SARS-CoV-2 disease outcomes (TRIM28, ACIN1, TNKS1BP1, HSPB1, and LARP7) (42). Additionally, TRIM28 deficiencies have been correlated with severe pediatric COVID-19 cases (43). These genes represent potential antiviral factors involved in SARS-CoV-2 infection control. Future work will determine the contributions of individual phosphorylation sites and answer whether the p38/MAPKs exert their effects on SARS-CoV-2 infection via a small number of potent substrates or the combined impact of many substrates with less potent effects.

## MATERIALS AND METHODS

### Cells

Human lung epithelial cell lines A549 (ATCC CCL-185) and Calu-3 (ATCC HTB-55); HEK 293T cells, a human kidney epithelial cell line (HEK 293T/17, ATCC CRL-11268); and Vero E6 cells (Vero 76, clone E6, Vero E6, ATCC CRL-1586), an African Green Monkey kidney epithelial cell line, were authenticated by ATCC (American Type Culture Collection). A monoclonal ACE2-expressing A549 cell line (A549-ACE2) was a kind gift from Brad Rosenberg. Monoclonal ACE2-expressing STAT1-knockout A549 cells (A549-ACE2ΔSTAT1) were generated as previously described (44, 45). All cell lines were cultured under humidified 5% CO_2_ conditions in 10% vol/vol fetal bovine serum (FBS, Thermo Fisher Scientific) and 100 I.U. penicillin and 100 µg/mL streptomycin (Pen/Strept, Corning) in Dulbecco’s Modified Eagle Medium (DMEM, Corning). Cells were confirmed negative for mycobacteria monthly (Lonza).

### Viruses

SARS-CoV-2 isolate USA-WA1/2020 (NR-52281) was obtained from BEI Resources, National Institute of Allergy and Infectious Diseases, and National Institutes of Health (NIH). Recombinant SARS-CoV-2 (rSARS-CoV-2), based on isolate USA-WA1/2020, and rSARS-CoV-2^N4A^ (S/T to A mutations at S21, S23, T24, and S26 on SARS-CoV-2 N) were generated as previously described (Ye et al., 2020). Virus stocks were grown by infecting Vero E6 cells in infection media (2% FBS, Pen/Strept, in DMEM) at an MOI of 0.01. Supernatant was collected 30 hours post-infection, concentrated through a 100-kDa centrifugal filter unit (Amicon), washed thrice in phosphate buffered saline (PBS), and further concentrated with a 100-kDa centrifugal filter unit (46). Virus stock titers were determined by plaque assay on Vero E6 cells. All work with live virus was done in the Centers for Disease Control and Prevention/US Department of Agriculture-approved biosafety level 3 (BSL-3) facility of the Icahn School of Medicine at Mount Sinai or the NYU Grossman School of Medicine in accordance with their respective guidelines for BSL-3 work.

### Cell treatment prior to harvest for mass spectrometry (MS)

**SB203580 pre-treatment**: Three plates each of 2 × 10^7^ A549-ACE2 cells in 15-cm plate format were treated with a final concentration of 1 μM SB203580 or 10 μM SB203580, and six plates were treated with an equal volume of DMSO as the drug-treated plates in 25 mL of infection media, and were incubated for 1 hour. All plates except three DMSO-treated plates (mock-infected) were infected by adding SARS-CoV-2 at an MOI of 0.1 directly to the drug-containing infection media. At 24-hour post-infection, all cells were lysed in urea lysis buffer as described below. **Terminal SB203580 treatment**: Eight plates of 2 × 10^7^ A549-ACE2 cells in 15-cm plate format were infected with SARS-CoV-2 at an MOI of 0.1 in 25 mL of infection media. An additional four plates were mock-infected in 25 mL of infection media. At 20-hour post-infection, half of the infected replicates were treated with SB203580 (Cell Signaling) in DMSO at a final concentration of 10 µM, and the other half of the infected replicates and all of the mock-infected replicates were treated with an equal volume of DMSO. Four hours after drug treatment, all cells were lysed in urea lysis buffer as described below. **siRNA knockdown**: Four plates each of 2 × 10^7^ A549-ACE2 cells in 15-cm plate format were transfected with pooled siRNAs against *MAPK11* or *MSK2* (Dharmacon), and eight plates were transfected with pooled siRNAs against NTC (Dharmacon) according to the manufacturing protocol for RNAiMAX (Thermo Fisher Scientific). At 48-hour post-transfection, the media on all plates was replaced with 25 mL of infection media. All plates except four NTC plates (mock-infected), were infected by adding SARS-CoV-2 at an MOI of 0.1 directly to the drug-containing infection media. At 36-hour post-infection, all cells were lysed in urea lysis buffer as described below.

### Cell lysis and digestion for mass spectrometry

Cells were washed twice in PBS. Cells were lysed in urea lysis buffer containing 8M urea, 100 mM ammonium bicarbonate (ABC), 150 mM NaCl, and a protease inhibitor and phosphatase inhibitor cocktail (HALT, Thermo Fisher Scientific). Lysates were probe-sonicated on ice three times for 1 second at 50% power, with 5 seconds of rest in between pulses. Protein content of the lysates was quantified using a micro BCA assay (Thermo Fisher Scientific). One milligram of protein per sample was treated with Tris-(2-carboxyethyl)phosphine to a final concentration of 4 mM and incubated at room temperature (RT) for 30 minutes. Iodoacetamide (IAA) was added to each sample to a final concentration of 10 mM, and samples were incubated in the dark at RT for 30 minutes. IAA was quenched by dithiothreitol to a concentration of 10 mM and incubated in the dark at RT for 30 minutes. Samples were then diluted with five sample volumes of 100 mM ABC. Trypsin Gold (Promega) was added at a 1:100 (enzyme:protein wt/wt) ratio and lysates were rotated for 16 hours at RT. 10% vol/vol trifluoroacetic acid (TFA) was added to each sample to a final concentration of 0.1% TFA. Samples were desalted under vacuum using Sep Pak tC18 cartridges (Waters). Each cartridge was first washed with 1 mL of 80% acetonitrile (ACN)/0.1% TFA, then washed three times with 1 mL of 0.1% TFA in H_2_O. Samples were then loaded on cartridges. Cartridges were washed three times with 1 mL of 0.1% TFA in H_2_O. Samples were then eluted with 1 mL of 40% ACN/0.1% TFA. Twenty micrograms of each sample was kept for protein abundance measurements, and the remainder was used for phosphopeptide enrichment. Samples were dried by vacuum centrifugation. Protein abundance samples were resuspended in 0.1% formic acid (FA) for mass spectrometry analysis.

### Phosphopeptide enrichment for mass spectrometry

For each sample batch and under vacuum, 500 µL (30 µL per sample) of 50% Ni-NTA Superflow bead slurry (QIAGEN) was added to a 2-mL empty spin column (Bio-Spin, Bio-Rad). Beads were washed three times with 1 mL of HPLC H_2_O, incubated four times with 1 mL of 100 mM EDTA for 30 seconds, washed three times with 1 mL of HPLC H_2_O, incubated four times with 1 mL of 15 mM FeCl_3_ for 1 minute, washed three times with 1 mL of HPLC H_2_O, and washed once with 1 mL of 0.5% vol/vol FA to remove residual iron. Beads were resuspended in 750 µL of 80% ACN/0.1% TFA. One milligram of digested peptides were resuspended in 83.33 µL of 40% ACN/0.1% TFA and 166.67 µL of 100% ACN/0.1% TFA, and 60 µL of the bead slurry was added to each sample and incubated for 30 minutes while rotating at RT. A C18 BioSPN column (Nest Group), centrifuged at 110× *g* for 1 minute for each step, was equilibrated two times with 200 μL of 80% ACN/0.1% TFA. Beads were loaded on the column and washed four times with 200 μL of 80% ACN/0.1% TFA, then washed three times with 200 μL of 0.5% FA. Then, 200 μL of 500 mM potassium phosphate buffer pH 7 was added three times to the column and incubated for 1 minute. Then, 200 μL of 0.5% FA was added three times to the column. Phosphopeptides were eluted two times with 100 μL of 40% ACN/0.1% FA and vacuum centrifuged to dryness. Phosphopeptides were resuspended in 25 µL of 4% FA/3% ACN for mass spectrometry analysis.

### Mass spectrometry data acquisition

All samples were analyzed on an Orbitrap Eclipse mass spectrometry system (Thermo Fisher Scientific) equipped with an Easy nLC 1200 ultra-high pressure liquid chromatography system (Thermo Fisher Scientific) interfaced via a Nanospray Flex nanoelectrospray source. For all analyses, samples were injected on a C18 reverse phase column (30 cm × 75 μm inner diameter) packed with ReprosilPur 1.9-µm particles). Mobile phase A consisted of 0.1% FA, and mobile phase B consisted of 0.1% FA/80% ACN. Peptides were separated by an organic gradient from 5% to 35% mobile phase B over 120 minutes followed by an increase to 100% B over 10 minutes at a flow rate of 300 nL/minute. Analytical columns were equilibrated with 6 µL of mobile phase A. To build a spectral library, samples from each set of biological replicates were pooled and acquired in data-dependent manner. Protein abundance samples were fractionated with Field Asymmetric Ion Mobility Spectrometry (FAIMS) fractionation with a FAIMS Pro device (Thermo Fisher Scientific). Each pooled sample was analyzed four times with four FAIMS compensation voltages (CVs) (−40 V, −55 V, −65 V, and −75 V). Data-dependent analysis (DDA) was performed by acquiring a full scan over an m/z range of 375–1,025 in the Orbitrap at 120,000 resolving power (@200 m/z) with a normalized AGC target of 100%, an RF lens setting of 30%, and an instrument-controlled ion injection time. Dynamic exclusion was set to 30 seconds, with a 10 ppm exclusion width setting. Peptides with charge states 2–6 were selected for MS/MS interrogation using higher energy collisional dissociation (HCD) with a normalized HCD collision energy of 28%, with 3 seconds of MS/MS scans per cycle. Similar settings were used for DDA of phosphopeptide-enriched pooled samples, with a dynamic exclusion of 45 seconds and no FAIMS fractionation. Data-independent analysis (DIA) was performed on all individual samples. An MS scan at 60,000 resolving power over a scan range of 390–1,010 m/z, an instrument-controlled AGC target, an RF lens setting of 30%, and an instrument-controlled maximum injection time, followed by DIA scans using 8 m/z isolation windows over 400–1,000 m/z at a normalized HCD collision energy of 28%.

### siRNA knockdown

2 × 10^4^ A549-ACE2 cells in a 96-well plate format were transfected (nine technical replicates), with 1 pmol siGENOME or ON TARGETplus siRNA pools (Dharmacon) prepared in 10 µL/replicate Opti-MEM (Corning) with a 1:3 ratio of siRNA:RNAiMax (Thermo Fisher Scientific). Forty-eight hours post-transfection, cells were infected with SARS-CoV-2 at an MOI of 0.1 or 2 in infection media. 8-, 30-, or 36 hours post-infection, supernatants were saved for plaque assay, one-third of replicates were fixed in 5% paraformaldehyde (PFA) in PBS for 24 hours, one-third of replicates were lysed in RIPA buffer with SDS (50 mM Tris HCl, 150 mM sodium chlorate, 1% vol/vol Triton X-100, 0.5% vol/vol sodium deoxycholate, 1% wt/vol sodium dodecyl sulfate (SDS), and protease inhibitors [MilliporeSigma]) and saved for western blotting, and the last third of replicates were lysed in RLT buffer (QIAGEN) and saved for RNA extraction and RT-qPCR analysis. For Calu-3 siRNA assays, 2 × 10^5^ cells in a 12-well plate format were transfected with 44 pmol of ON TARGETplus siRNA with lipofectamine RNAimax according to manufacturer’s instructions. Seventy-two hours post-transfection, cells were washed and lysed in RIPA buffer with SDS for western blotting, or cells were infected with SARS-CoV-2 at an MOI of 0.15 in infection media. Twenty-four hours post-infection, cells were washed and fixed in 5% PFA for 15 minutes for SARS-CoV-2 N staining and flow cytometry analysis.

### Immunofluorescence assay

Fixed cells were washed with PBS and permeabilized for 10 minutes in 0.2% vol/vol Triton X-100 in PBS. Cells were incubated in blocking buffer (3% wt/vol bovine serum albumin [BSA], 0.1% vol/vol Triton X-100, 0.2% wt/vol fish gelatin in PBS) at RT for 1 hour. Cells were incubated in primary antibody (1:1,000 mouse anti-SARS-CoV-1/2 N 1C7C7 antibody, a kind gift from Thomas Moran) in antibody buffer (1% wt/vol BSA, 0.03% vol/vol Triton X-100, 0.1% fish gelatin in PBS) overnight at 4°C. Cells were washed thrice in PBS. Cells were incubated in 1:1,000 anti-mouse AlexaFluor488 or anti-mouse AlexaFluor594 (Thermo Fisher Scientific) and 4′,6-diamidino-2-phenylindole counterstain (DAPI, Thermo Fisher Scientific) in antibody buffer at RT for 1 hour. Cells were washed thrice in PBS and imaged in 100 µL PBS on a Celígo Imaging Cytometer (Nexcelcom Bioscience) or a CX7 Imaging Cytometer (Thermo Fisher Scientific). Celígo software or CX7 software was used to quantify the total number of cells by DAPI nuclear staining and the number of N-positive cells by N staining.

### Plaque assay

Twenty-five microliters of virus-containing supernatant was serially 10-fold diluted in infection media. Inoculum of 100 µL was added to confluent Vero E6 cells in a 24-well plate format and incubated at RT for 1 hour and agitated often to avoid drying. One milliliter of semi-solid overlay (0.1% wt/vol agarose, 4% vol/vol FBS, and Pen/Strept in DMEM) was added to each well, and plates were incubated at 37°C for 48 hours. Cells were fixed in 5% PFA in PBS for 24 hours at RT. Cells were washed twice with water. Cells were incubated in 0.5 mL staining dye (2% wt/vol crystal violet, 20% vol/vol ethanol in water) for 10 minutes at RT. Stained cells were washed with water and allowed to dry before plaques were counted, and plaque-forming units per milliliter was calculated as follows: [(No. of plaques)/(mL of inoculum*10^dilution factor^)].

### Western blotting

Lysates were incubated at 95°C for 10 minutes in Laemmli sample buffer (Bio-Rad Laboratories). Lysates were run on an SDS-PAGE gel with a protein ladder standard (Bio-Rad Laboratories) and transferred to a nitrocellulose membrane (Bio-Rad Laboratories). Blots were incubated in 5% milk in TBST for 1 hour at RT. Blots were incubated in primary antibody (1:1,000 anti-MAPK11 Cell Signaling no. 2339, 1:1,000 anti-MAPK14 Cell Signaling no. 8690, 1:1,000 anti-MAPK12 Cell Signaling no. 2307, 1:1,000 anti-MSK2 Cell Signaling no. 3679, 1:5,000 anti-SARS-CoV-1/2 N (1 g/mL, clone 1C7C7, a kind gift from Thomas Moran), 1:3,000 anti-β-actin Cell Signaling no. 3700, 1:1,000 anti-vinculin Cell Signaling no. 13901, 1:1,000 anti-MX1 abcam no. ab95926, 1:1,000 anti-P(Y701)-STAT1 Cell Signaling no. 9167, 1:1,000 anti-STAT1 Cell Signaling no. 14995) in 1% milk in TBST overnight at 4°C. Blots were washed thrice for 5 minutes in TBST. Blots were incubated in secondary HRP-conjugated (Bio-Rad Laboratories) or infrared-conjugated secondary antibodies (LICOR Biosciences) in 1% milk in TBST. Blots were washed thrice for 5 minutes in TBST. Blots were imaged on a Chemiluminescence digital imager (Bio-Rad Laboratories) using FEMTO ECL reagent (Thermo Fisher Scientific) or an infrared digital imager (LICOR Biosciences).

### Flow cytometry analysis of SARS-CoV-2-infected Calu-3

Fixed cells were washed with PBS and incubated for 15 minutes in intracellular staining buffer (PBS with 0.2% BSA and 0.05% saponin). Cells were incubated in primary antibody (1:1,000 anti-SARS-CoV-1/2 N, clone 1C7C7) in a staining buffer for 1 hour. Cells were washed thrice in staining buffer and incubated in 1 g/mL (1:1,000) secondary antibody goat anti-mouse PE (Thermo Fisher Scientific) in staining buffer at RT for 1 hour. Finally, cells were washed twice in staining buffer and resuspended in PBS to score N-positive cells on an Attune NxT flow cytometer (Thermo Fisher Scientific).

### Cell viability assay

2 × 10^4^ A549-ACE2 cells in a 96-well white-bottom plate were transfected in triplicate with siRNA pools. Seventy-two hours post-transfection, the plate was equilibrated to RT for 30 minutes. For Calu-3 cells, 2 × 10^5^ cells in a 12-well plate format were transfected in triplicate with siRNA pools for 72 hours. Titerglo buffer of 100 or 200 µL with substrate (Promega Corporation) was added to each well for A549-ACE2 cells or Calu-3 cells, respectively. Plate was nutated at RT for 2 minutes to lyse the cells. Plate was incubated at RT for 10 minutes. For Calu-3 cells, 10 µL of cell lysates was transferred in a 96-well white-bottom plate cells. Plates were read for luminescence end-point kinetics with a 1s integration on a Cytation 5 Plate Reader using Gen5 software (BioTek Instruments). Relative luminescence units for each siRNA condition were normalized to NTC siRNA and displayed as a percentage.

### Entropy of N analysis

Entropy values for each amino acid on N for SARS-CoV-2 sequences from GISAID (Global Initiative on Sharing All Influenza Data) were downloaded from Next Strain on December 13, 2021 (31).

### mRNA sequencing and analysis

2 × 10^5^ A549-ACE2 cells were transfected with siRNA pools as previously indicated. Forty-eight hours post-transfection, cells were infected with SARS-CoV-2 at an MOI of 0.75 in infection media. Eight hours post-infection, cells were lysed in 1 mL of Trizol (Invitrogen). RNA was extracted and DNase I was treated using Direct-zol RNA Miniprep kit (Zymo Research) according to the manufacturer’s protocol. RNA-Seq libraries of polyadenylated RNA were prepared with the TruSeq RNA Library Prep Kit v2 (Illumina) according to the manufacturer’s protocols. Libraries were sequenced with an Illumina NextSeq 500 platform. Raw sequencing reads were aligned to the hg19 human genome with the Basespace RNA-Seq Alignment application (Illumina). GO-term enrichment was performed using Biojupies (47). Alignment to viral genomes was performed using bowtie2 (48). The SARS-CoV-2 USA/WA1/2020 strain genome was used for analysis in this study (GenBank: MN985325). Gene set enrichment analysis was performed with the enrichr package in R (49). Genome coverage (viral gene counts at each nucleotide position) was analyzed using Integrative Genomics Viewer and visualized with ggplot2 (50). All raw and processed mRNA-Seq data are available on NCBI GEO GSE183999 (https://www.ncbi.nlm.nih.gov/geo/geo2r/?acc=GSE183999).

### RNA extraction and RT-qPCR analysis

Cells were lysed in RLT buffer and RNA was extracted using an RNeasy 96 kit (QIAGEN) according to the manufacturer’s protocol. One-step RT-qPCR was performed on 2 µL of RNA using the Luna Universal One-Step RT-qPCR Kit (NEB Biosciences) and primers for β-tubulin (Forward: 5′-GCCTGGACCACAAGTTTGAC-3′; Reverse: 5′-TGAAATTCTGGGAGCATGAC-3′), SARS-CoV-2 *NSP14* (Forward: 5′-TGGGGYTTTACRGGTAACCT-3′; Reverse: 5′-AACRCGCTTAACAAAGCACTC-3′), and *TRS-N* (Forward: 5′-CTCTTGTAGATCTTCTCTAAACGAAC-3′; Reverse: 5′-GGTCCACCAAACGTAATGCG-3′) as previously described (44, 51). Reactions were analyzed on a Lightcycler 480 II Instrument (Roche). ΔΔ cycle threshold values were calculated relative to mock-infected samples and NTC samples.

### Statistical analysis

All experiments were performed in at least biological triplicate with at least three technical replicates per biological replicate, when appropriate. Biological replicates are defined here as separate wells or plates of an experiment. Technical replicates are defined here as separate instrumental measurements within a single biological replicate. All experiments, with the exception of the mass spectrometry and RNA-Seq experiments due to the extensive sample processing, were performed at least separate times with separate passages of cells on separate days to ensure results were consistent. Unless otherwise noted, error bars indicate 1 SD from the mean of three biological replicates. Unless otherwise noted, one-way analysis of variance (ANOVA) tests with *post hoc* testing by Tukey’s method (in comparison to control) were performed to generate *P*-values in R with the rstatix package (Kassambara, 2019). “****” = *P*-value < 0.0001, “***” = 0.0001 < *P*-value < 0.001, “**” = 0.001 < *P*-value < 0.01, “*” = 0.01 < *P*-value < 0.05, “NS” = *P*-value > 0.05.

### Mass spectrometry data analysis

All raw mass spectrometry data generated in this study were analyzed by the Spectronaut software suite (Biognosys) (52). DDA-MS data were analyzed to build a spectral library by searching against a protein sequence database composed of SwissProt human sequences (downloaded on 10 October 10, 2019) and SARS-CoV-2 strain USA/WA1/2020 sequences using Biognosys factory settings, which considered a static modification for cysteine carbamidomethylation and variable modifications for methionine oxidation and protein *N*-terminal acetylation. We added variable modifications for serine/threonine/tyrosine phosphorylation for phospho-enriched samples. All DDA-MS runs generated in this study were combined to make one spectral library against which all DIA-MS data were analyzed. DIA-MS data were also analyzed by Spectronaut to extract fragment ion intensity information based on the spectral library described above using Biognosys factory settings. The data were exported as a tab-delimited, MSstats-formatted report. Spectronaut reports were analyzed by the MSstats package in the Rstudio statistical programming environment (53). Before MSstats analysis, protein group accessions were converted to phosphosite groups with a Perl script. “Phosphosite group” refers to modified residues identified on peptides with sequences that are unique for a single protein or shared across a group of homologous proteins. Phosphosite groups also separate phosphosites identified on singly, doubly, or triply phosphorylated peptides. Data were processed by MSstats to equalize medians, summarize features using Tukey’s median polish, and impute missing values by accelerated failure model. Intensities below the 0.999 quantile were considered missing values. Principal component analysis was performed on MSstats estimated intensities using the prcomp function in R, and the first two principal components were plotted for all data sets. Sample correlation analysis was performed by pairwise Pearson correlation coefficient calculation in R.

### Gene ontology enrichment and kinase activity analyses

GO enrichment and kinase activity analyses were performed using the GSEA method with the fgsea package in R (24). For kinase activity analysis, kinase substrate interactions were derived from the PhosphositePlus Kinase Substrate Dataset (23). Protein substrates of each kinase were compiled into gene sets. For GO enrichment analysis, GO terms and definitions were downloaded from the GO resource (downloaded on February 18, 2021) and genes within each GO term were grouped as gene sets. For both GO enrichment analysis and kinase activity analysis, for each comparison considered, the data were ranked by log_2_(fold-change) and subjected to fgsea testing using the gene sets described above.

### Unbiased identification of p38β substrates

For hierarchical clustering, comparisons indicated in Fig. 5 were first filtered for missing log­fold-change values in any comparison. Distances were calculated based on Euclidean distance using the dist function in R, and the data were hierarchically ordered using the hclust function in R. Next, the data were divided into *n* clusters in decreased order of dendrogram height in 99 iterations with *n* ranging from 2 to 100 using the cutree function in R. For each iteration, the enrichment of annotated p38α/β substrates within each cluster was calculated using a hypergeometric test with the dhyper function in R. *P*-values were adjusted by Benjamini–Hochberg method with the p.adjust function in R. Annotations of p38α/β substrates were derived from the Phosphosite Plus Kinase Substrate data set (23). Phosphosites in the cluster with the minimum hypergeometric *P*-value in all iterations across all clusters comprise our putative p38β substrates.

## Data Availability

Raw mass spectrometry data have been deposited to the ProteomeXchange Consortium via the PRIDE partner repository with the data set identifier PXD035451.

## SUPPLEMENTAL MATERIAL

Supplemental figures mbio.01007-23-s0001.pdf Figures S1-S6.

Table S1 mbio.01007-23-s0002.xlsx Combined file of proteomics results.

Table S2 mbio.01007-23-s0003.xlsx Log2fold-change values compiled across published SARS-CoV-2 phosphoproteomics studies.

Table S3 mbio.01007-23-s0004.xlsx Kinase analysis results related to Fig. 1.

Table S4 mbio.01007-23-s0005.xlsx Differentially expressed genes from RNAseq analysis related to Fig. 3.

Table S5 mbio.01007-23-s0006.xlsx Gene ontology enrichment analysis of RNAseq data related to Fig. 3.

Table S6 mbio.01007-23-s0007.xlsx Gene ontology enrichment analysis of proteomics data related to Fig. 4.

Table S7 mbio.01007-23-s0008.xlsx Log2fold-change values for phosphorylation sites in a p38 cluster related to Fig. 5.

ASM does not own the copyrights to Supplemental Material that may be linked to, or accessed through, an article. The authors have granted ASM a non-exclusive, world-wide license to publish the Supplemental Material files. Please contact the corresponding author directly for reuse.
