# Supplementary material for: SARS-CoV-2 hijacks p38β/MAPK11 to promote virus replication
Source: mBio. 2023 Jun 22;14(4):e01007-23. doi: 10.1128/mbio.01007-23 (PMC10470746; doi:10.1128/mbio.01007-23)
Supplement: Supplemental figures — Figures S1-S6. [file mbio.01007-23-s0001.pdf]

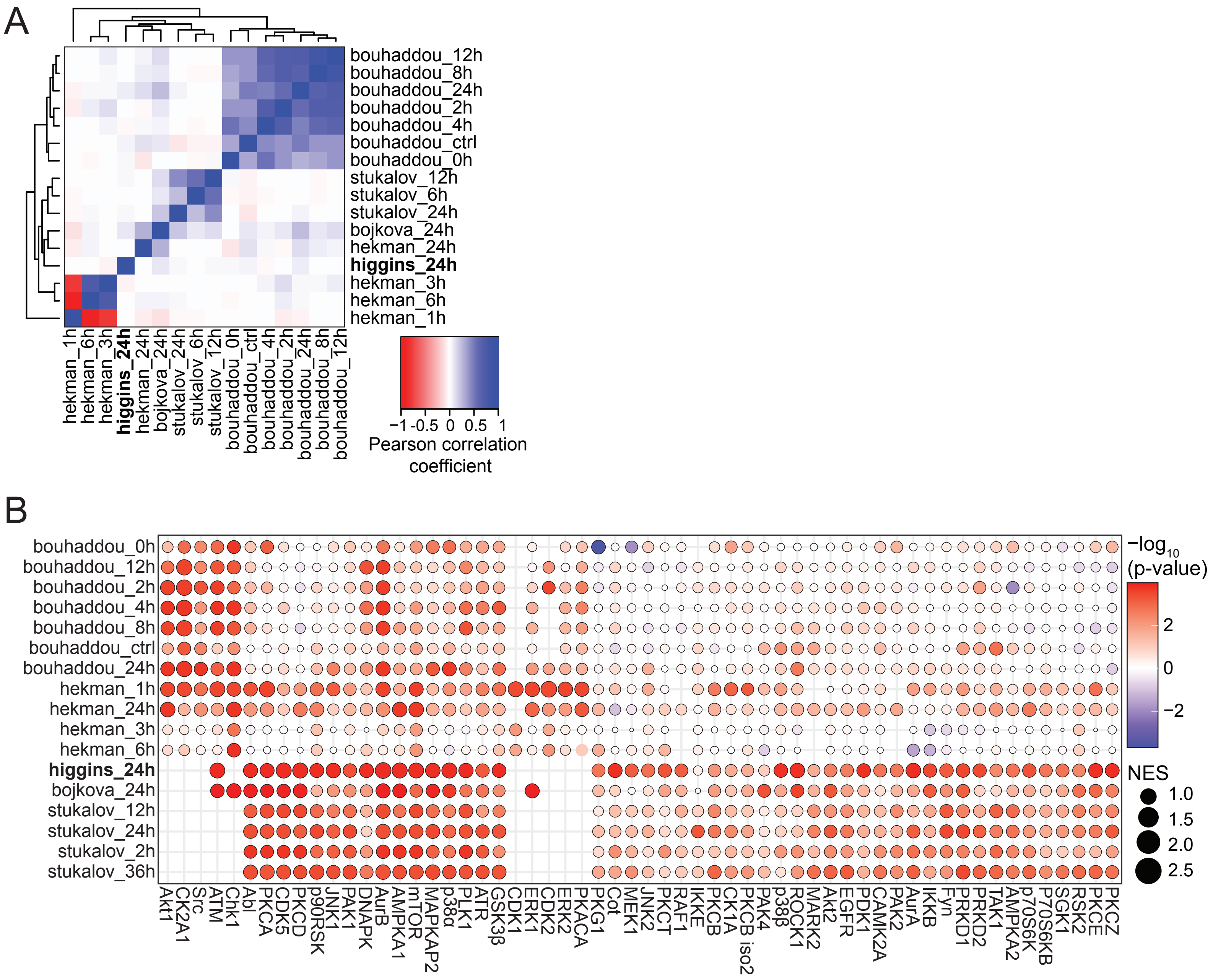

Figure S1: A) Heatmap of pairwise Pearson coefficients for protein group log2(fold-change) profiles from this study and published studies indicated; B) Bubble plot of kinase activity analysis of phosphosite group log2(fold-change) profiles from this study and published studies indicated; the absolute value of the normalized enrichment score (NES) is indicated by node sizes and the -log10(p-value) is indicated by the color scale.

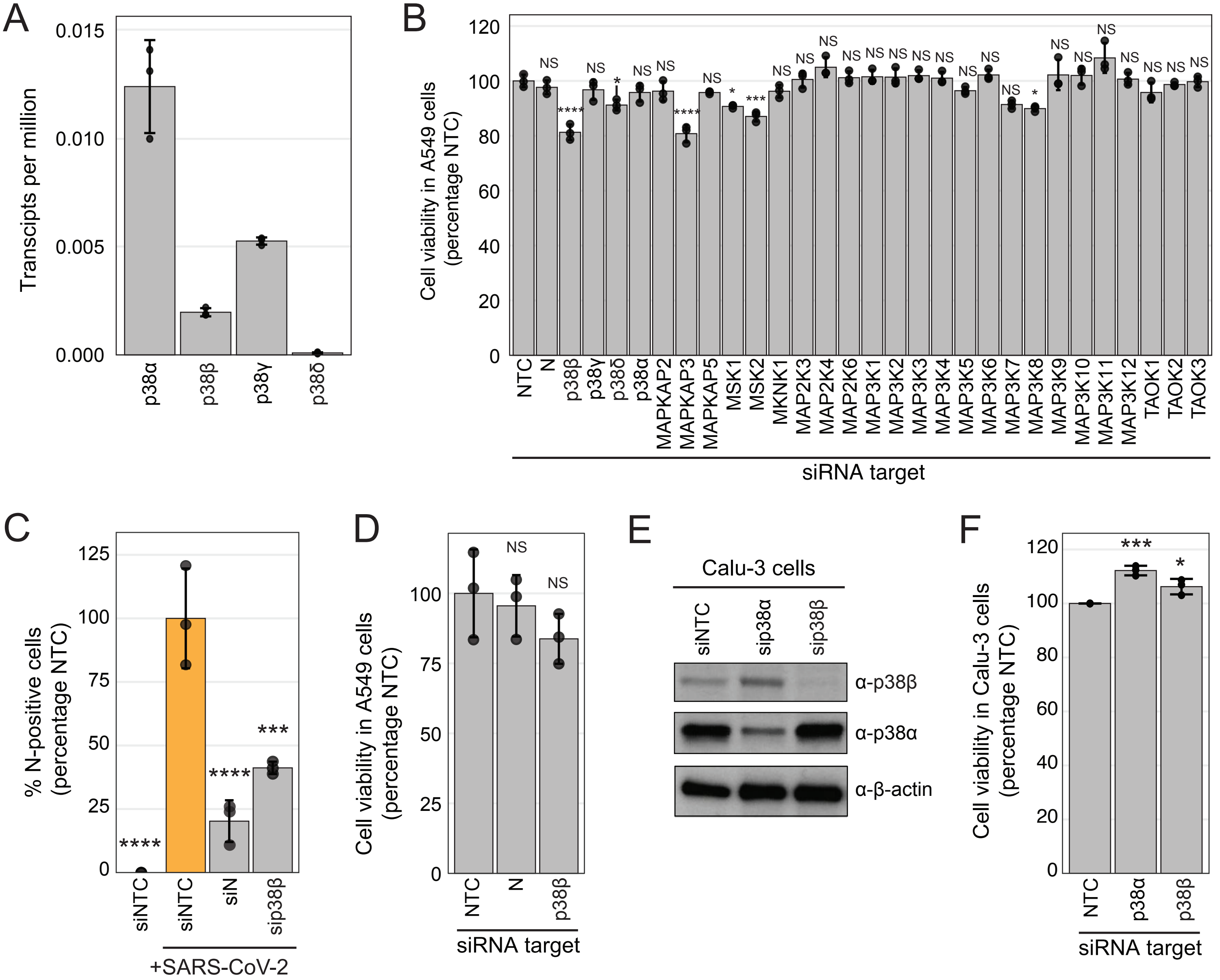

Figure S2: A) Plot of transcripts per million for each indicated p38 isoform from mRNA-sequencing performed on A549-ACE2 cells; B) Plot of A549-ACE2 cell viability normalized to siNTC transfection; C) Plot of the percent of SARS-CoV-2 N-positive cells analyzed using immunofluorescence cytometry, represented as a percentage compared to the infected control condition, for Dharmacon ON-TARGET-plus siRNA pools targeting NTC or sip38β, (different from Dharmacon siGENOME siRNA pools used in Figure 2B), after SARS-CoV-2 infection at an MOI of 0.1 for 30h in A549-ACE2 cells; D) Plot of A549-ACE2 cell viability normalized to siNTC for siRNA pools used in S2C; E) Western blot of Calu-3 cell lysates collected in parallel with cells from 2F; F) Plot of Calu-3 cell viability normalized to siNTC; all error bars represent one standard deviation from the mean for three biological replicates; p-value annotations were calculated using a one-way ANOVA test with post hoc testing using Tukey's method comparing each condition to siNTC for three biological replicates; "****" = p-value < 0.0001, "***" = 0.0001 < p-value < 0.001, "**" = 0.001 < p-value < 0.01, "*" = 0.01 < p-value < 0.05, "NS" = p-value > 0.05

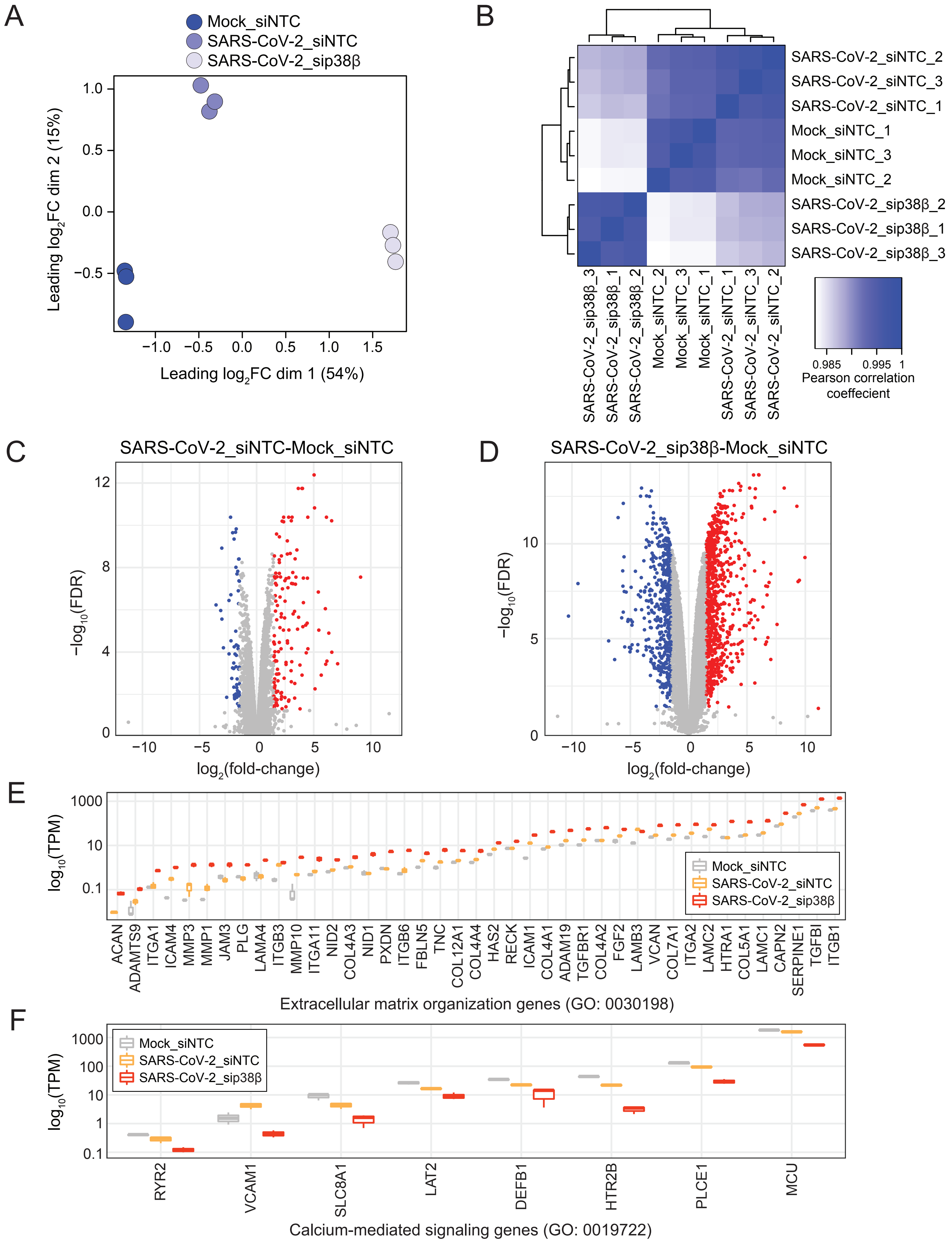

A
Mock_siNTC
SARS-CoV-2_siNTC
SARS-CoV-2_sip38β
Leading log2FC dim 2 (15%)
Leading log2FC dim 1 (54%)
B
SARS-CoV-2_siNTC_2
SARS-CoV-2_siNTC_3
SARS-CoV-2_siNTC_1
Mock_siNTC_1
Mock_siNTC_3
Mock_siNTC_2
SARS-CoV-2_sip38β_2
SARS-CoV-2_sip38β_1
SARS-CoV-2_sip38β_3
Pearson correlation coeffecient
C
SARS-CoV-2_siNTC-Mock_siNTC
−log10(FDR)
log2(fold-change)
D
SARS-CoV-2_sip38β-Mock_siNTC
−log10(FDR)
log2(fold-change)
E
log10(TPM)
Mock_siNTC
SARS-CoV-2_siNTC
SARS-CoV-2_sip38β
ACAN ADAMTS9 ITGA1 ICAM4 MMP3 MMP1 JAM3 PLG LAMA4 ITGB3 MMP10 ITGA11 NID2 COL4A3 NID1 PXDN ITGB6 FBLN5 TNC COL12A1 COL4A4 HAS2 RECK ICAM1 COL4A1 ADAM19 TGFBR1 COL4A2 FGF2 LAMB3 VCAN COL7A1 ITGA2 LAMC2 HTRA1 COL5A1 LAMC1 CAPN2 SERPINE1 TGFBI ITGB1
Extracellular matrix organization genes (GO: 0030198)
F
log10(TPM)
Mock_siNTC
SARS-CoV-2_siNTC
SARS-CoV-2_sip38β
RYR2 VCAM1 SLC8A1 LAT2 DEFB1 HTR2B PLCE1 MCU
Calcium-mediated signaling genes (GO: 0019722)

Figure S3: A) Plot of principal component analysis of mRNA-Seq samples; B) Heatmap of Pearson correlation analysis of mRNA-Seq samples; C-D) Volcano plot of differentially expressed genes for the indicated condition comparisons; grey is grey is not differentially expressed, red is upregulated and blue is downregulated; E-F) Plot of log10(transcripts per million) for each gene represented in the indicated GO term for each condition, from same analysis as 3D.

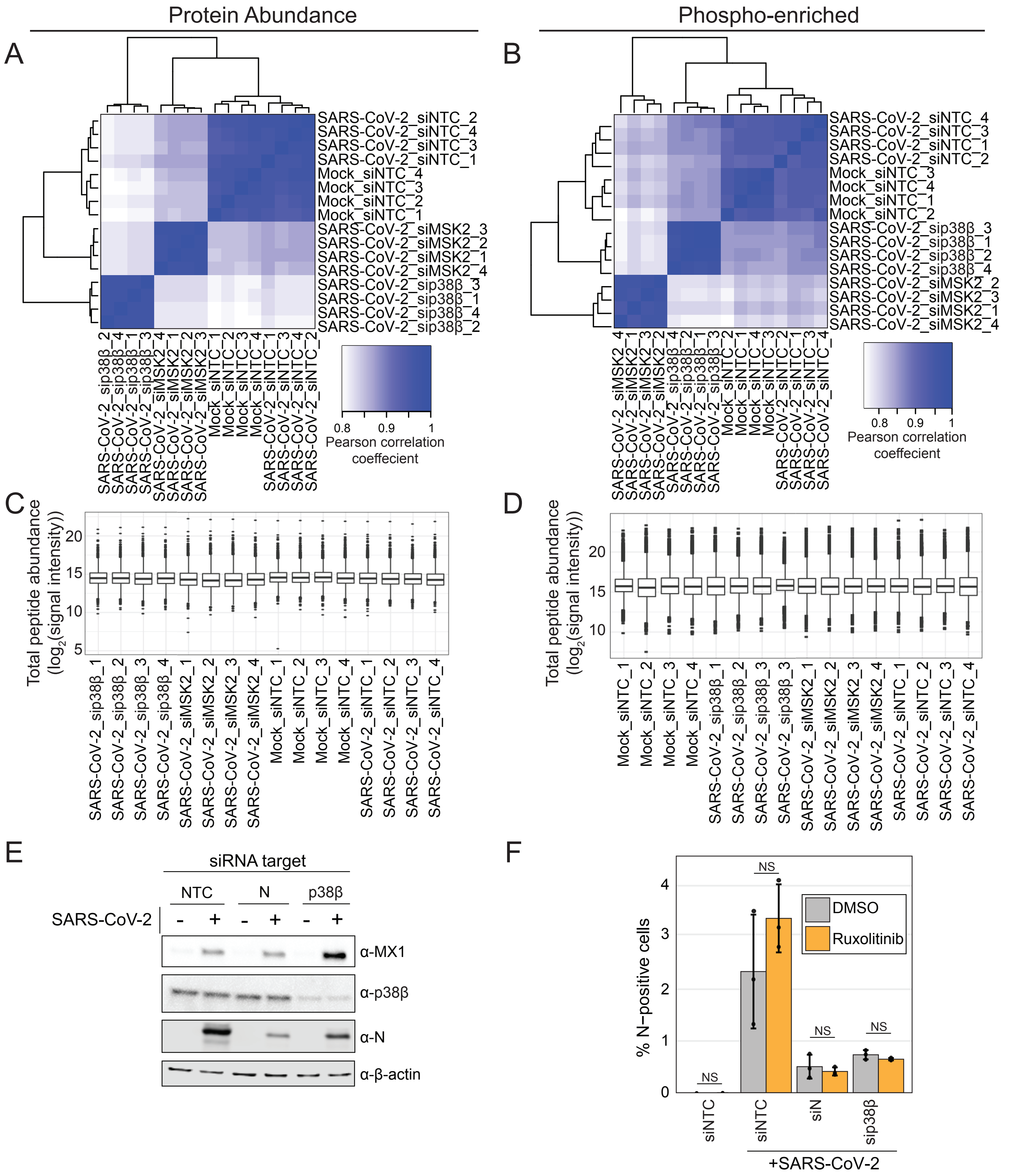

Figure S4: A) Heatmap of Pearson's correlation analysis of protein abundance mass spectrometry samples; B) Heatmap of Pearson's correlation analysis of phosphopeptide-enriched mass spectrometry samples; C) Plot of peptide abundance (mass spectrometry signal intensity) for each biological replicate of protein abundance samples; D) Plot of phosphopeptide abundance (mass spectrometry signal intensity) for each biological replicate of phosphopeptide-enriched samples; E) Western blot of lysates from cells transfected with siRNA targeting each indicated gene and infected with SARS-CoV-2 MOI 0.1 or mock-infected for 30h in A549-ACE2 cells; F) Plot of the percent of SARS-CoV-2 N-positive cells analyzed using immunofluorescence cytometry for each indicated transfection condition after SARS-CoV-2 infection at an MOI of 0.1 for 30h in A549-ACE2 cells in the presence of DMSO or ruxolitinib; error bars represent one standard deviation from the mean for three biological replicates; p-values were calculated using a one-way ANOVA test with post hoc testing using Tukey's method comparing each condition between each cell type for three biological replicates; "NS" = p-value > 0.05.

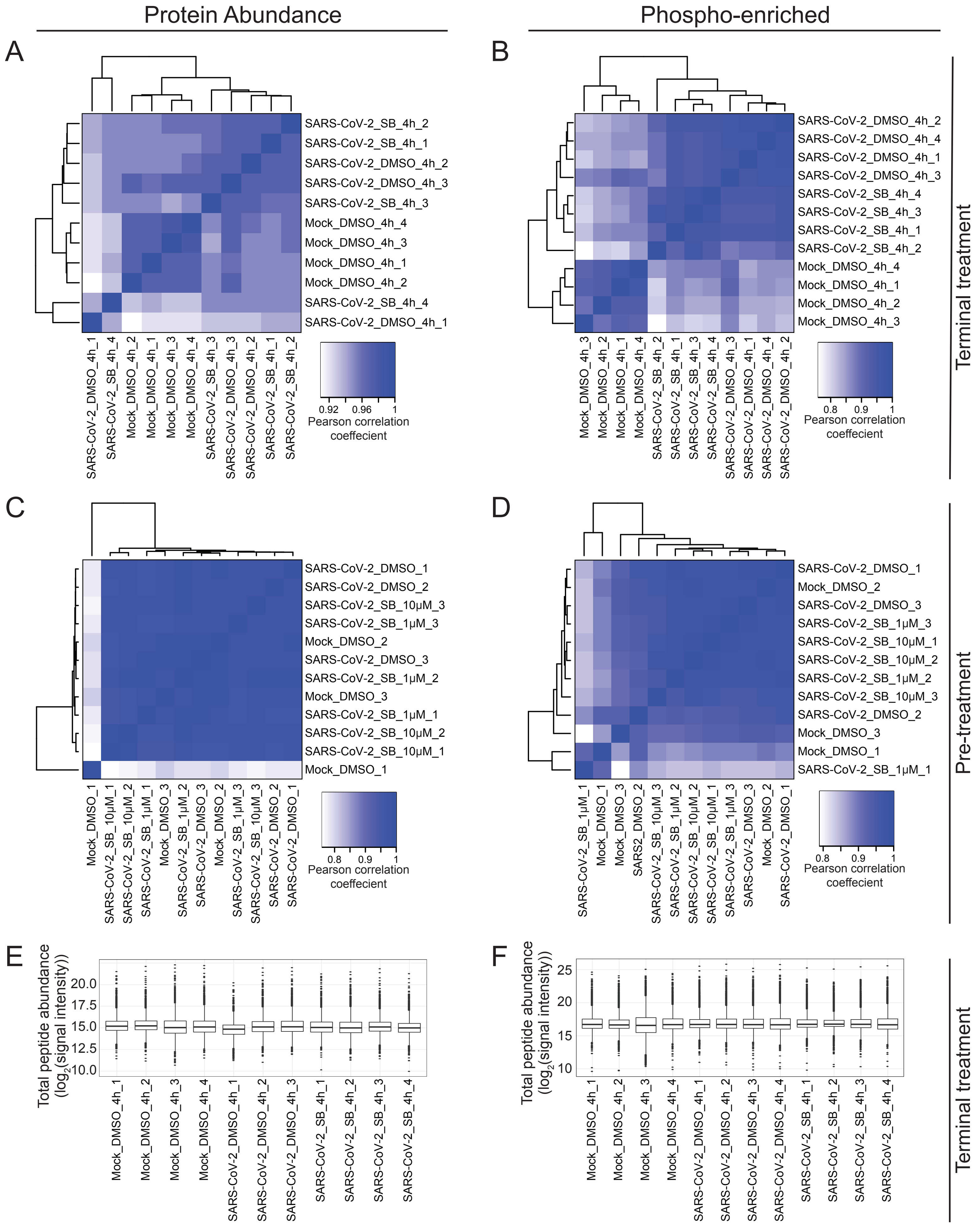

Protein Abundance
Phospho-enriched
A
B
C
D
E
F
Terminal treatment
Pre-treatment
Terminal treatment
SARS-CoV-2_SB_4h_2
SARS-CoV-2_SB_4h_1
SARS-CoV-2_DMSO_4h_2
SARS-CoV-2_DMSO_4h_3
SARS-CoV-2_SB_4h_3
Mock_DMSO_4h_4
Mock_DMSO_4h_3
Mock_DMSO_4h_1
Mock_DMSO_4h_2
SARS-CoV-2_SB_4h_4
SARS-CoV-2_DMSO_4h_1
0.92 0.96 1
Pearson correlation coeffecient
SARS-CoV-2_DMSO_4h_2
SARS-CoV-2_DMSO_4h_4
SARS-CoV-2_DMSO_4h_1
SARS-CoV-2_DMSO_4h_3
SARS-CoV-2_SB_4h_4
SARS-CoV-2_SB_4h_3
SARS-CoV-2_SB_4h_1
SARS-CoV-2_SB_4h_2
Mock_DMSO_4h_4
Mock_DMSO_4h_1
Mock_DMSO_4h_2
Mock_DMSO_4h_3
0.8 0.9 1
Pearson correlation coeffecient
SARS-CoV-2_DMSO_1
SARS-CoV-2_DMSO_2
SARS-CoV-2_SB_10μM_3
SARS-CoV-2_SB_1μM_3
Mock_DMSO_2
SARS-CoV-2_DMSO_3
SARS-CoV-2_SB_1μM_2
Mock_DMSO_3
SARS-CoV-2_SB_1μM_1
SARS-CoV-2_SB_10μM_2
SARS-CoV-2_SB_10μM_1
Mock_DMSO_1
0.8 0.9 1
Pearson correlation coeffecient
SARS-CoV-2_DMSO_1
Mock_DMSO_2
SARS-CoV-2_DMSO_3
SARS-CoV-2_SB_1μM_3
SARS-CoV-2_SB_10μM_1
SARS-CoV-2_SB_10μM_2
SARS-CoV-2_SB_1μM_2
SARS-CoV-2_SB_10μM_3
SARS-CoV-2_DMSO_2
Mock_DMSO_3
Mock_DMSO_1
SARS-CoV-2_SB_1μM_1
SARS2_DMSO_2
0.8 0.9 1
Pearson correlation coeffecient
Total peptide abundance (log2(signal intensity))
20.0
17.5
15.0
12.5
10.0
25
20
15
10
Mock_DMSO_4h_1
Mock_DMSO_4h_2
Mock_DMSO_4h_3
Mock_DMSO_4h_4
SARS-CoV-2_DMSO_4h_1
SARS-CoV-2_DMSO_4h_2
SARS-CoV-2_DMSO_4h_3
SARS-CoV-2_DMSO_4h_4
SARS-CoV-2_SB_4h_1
SARS-CoV-2_SB_4h_2
SARS-CoV-2_SB_4h_3
SARS-CoV-2_SB_4h_4

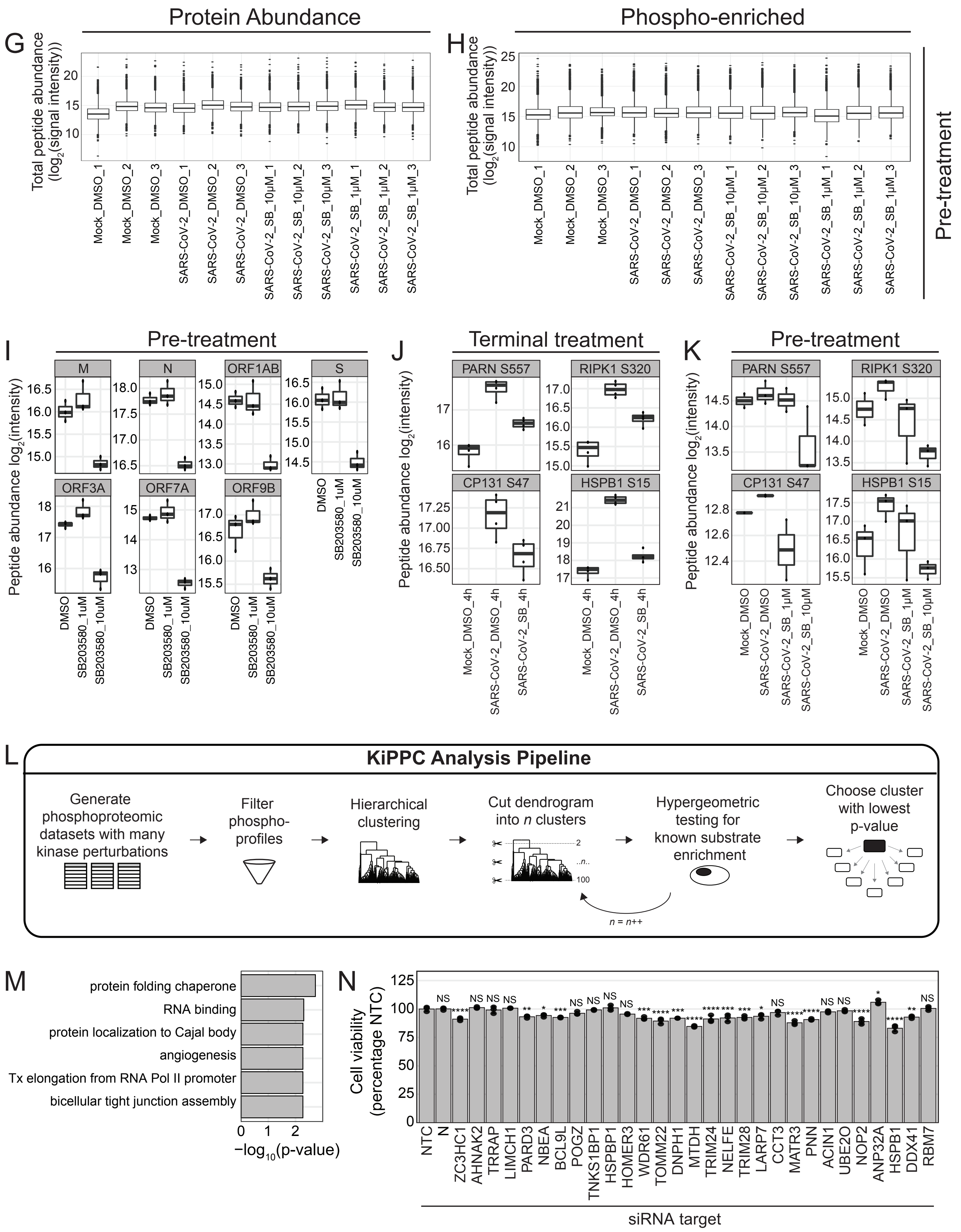
G
Protein Abundance
Total peptide abundance (log2(signal intensity))
Mock_DMSO_1
Mock_DMSO_2
Mock_DMSO_3
SARS-CoV-2_DMSO_1
SARS-CoV-2_DMSO_2
SARS-CoV-2_DMSO_3
SARS-CoV-2_SB_10μM_1
SARS-CoV-2_SB_10μM_2
SARS-CoV-2_SB_10μM_3
SARS-CoV-2_SB_1μM_1
SARS-CoV-2_SB_1μM_2
SARS-CoV-2_SB_1μM_3
H
Phospho-enriched
Total peptide abundance (log2(signal intensity))
Pre-treatment
I
Pre-treatment
Peptide abundance log2(intensity)
M
N
ORF1AB
S
ORF3A
ORF7A
ORF9B
DMSO
SB203580_1uM
SB203580_10uM
J
Terminal treatment
Peptide abundance log2(intensity)
PARN S557
RIPK1 S320
CP131 S47
HSPB1 S15
Mock_DMSO_4h
SARS-CoV-2_DMSO_4h
SARS-CoV-2_SB_4h
K
Pre-treatment
Peptide abundance log2(intensity)
PARN S557
RIPK1 S320
CP131 S47
HSPB1 S15
Mock_DMSO
SARS-CoV-2_DMSO
SARS-CoV-2_SB_1μM
SARS-CoV-2_SB_10μM
L
KiPPC Analysis Pipeline
Generate phosphoproteomic datasets with many kinase perturbations
Filter phospho-profiles
Hierarchical clustering
Cut dendrogram into n clusters
2
..n..
100
n = n++
Hypergeometric testing for known substrate enrichment
Choose cluster with lowest p-value
M
protein folding chaperone
RNA binding
protein localization to Cajal body
angiogenesis
Tx elongation from RNA Pol II promoter
bicellular tight junction assembly
−log10(p-value)
N
Cell viability (percentage NTC)
NTC
N
ZC3HC1
AHNAK2
TRRAP
LIMCH1
PARD3
NBEA
BCL9L
POGZ
TNKS1BP1
HSPBP1
HOMER3
WDR61
TOMM22
DNPH1
MTDH
TRIM24
NELFE
TRIM28
LARP7
CCT3
MATR3
PNN
ACIN1
UBE2O
NOP2
ANP32A
HSPB1
DDX41
RBM7
siRNA target

Figure S5: A-B) Heatmap of Pearson's correlation analysis of protein abundance (A) or phospho-enriched (B) mass spectrometry samples from "terminal treatment" experiment arm; C-D) Heatmap of Pearson's correlation analysis of protein abundance (A) or phospho-enriched (B) mass spectrometry samples from "pre-treatment" experiment arm; E-F) Plot of peptide abundance (log2(mass spectrometry signal intensity)) for each replicate of protein abundance (E) or phospho-enriched (F) samples from "terminal treatment" experiment arm; E-F) Plot of peptide abundance (log2(signal intensity)) for each replicate of protein abundance (G) or phospho-enriched (H) samples from "pre-treatment" experiment arm; I) Plot of log2(signal intensity)of known p38β substrates from terminal-treatment experiment for each comparison; J) Plot log2(signal intensity) of known p38 β substrates from pre-treatment experiment for each comparison; K) Plot log2(signal intensity) of detected SARS-CoV-2 proteins from pre-treatment experiment for each comparison; L) Schematic of kinase perturbation phospho-profile clustering (KiPPC) pipeline; M) Gene ontology terms enriched from cluster-of-interest proteins using GSEA; N) Plot of A549-ACE2 cell viability normalized to siNTC for each cluster-of-interest-gene siRNA transfection; error bars represent one standard deviation from the mean for three biological replicates; p-values were calculated using a one-way ANOVA test with post hoc testing using Tukey's method comparing each condition to the infected control condition, for three biologicals replicates; "****" = p-value < 0.0001, "***" = 0.0001 < p-value < 0.001, "**" = 0.001 < p-value < 0.01, "*" = 0.01 < p-value < 0.05, "NS" = p-value > 0.05.

A

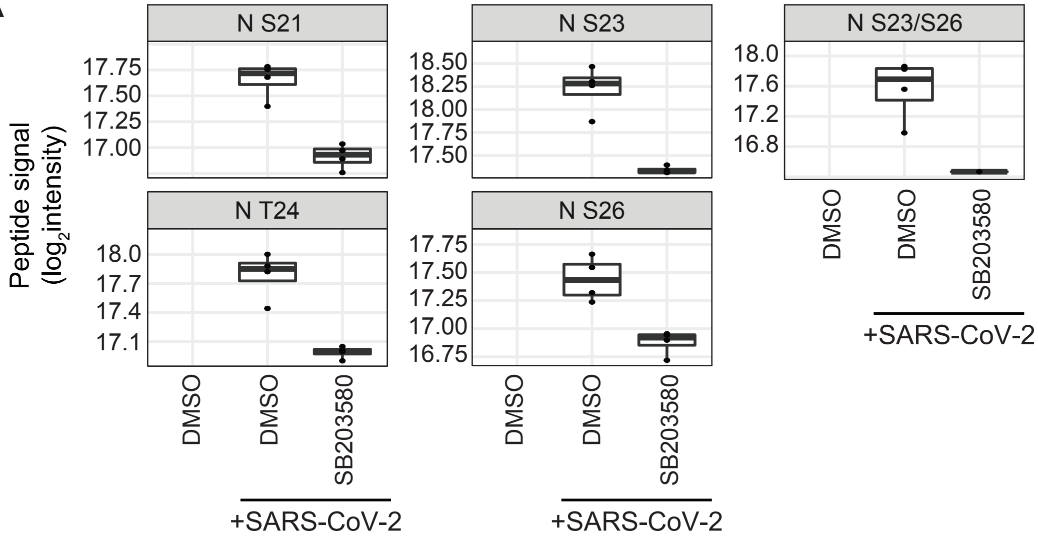

Figure S6: A) Plot of log2(signal intensity) of each significantly differentially abundant phosphosite group on SARS-CoV-2 N from the terminal-treatment experiment arm (Figure 5A).
